# Techniques for Interface Stress Measurements within Prosthetic Sockets of Transtibial Amputees: A Review of the Past 50 Years of Research

**DOI:** 10.3390/s16071119

**Published:** 2016-07-20

**Authors:** Ebrahim A. Al-Fakih, Noor Azuan Abu Osman, Faisal Rafiq Mahmad Adikan

**Affiliations:** 1 Department of Biomedical Engineering, Faculty of Engineering, University of Malaya, Kuala Lumpur 50603, Malaysia; azuan@um.edu.my; 2 Department of Electrical Engineering, Faculty of Engineering, University of Malaya, Kuala Lumpur 50603, Malaysia; rafiq@um.edu.my

**Keywords:** prosthetic sockets, transtibial amputee, pressure measurement transducers, interface stress investigations, PTB sockets, TSB sockets, liners, suspension systems, biomechanics

## Abstract

The distribution of interface stresses between the residual limb and prosthetic socket of a transtibial amputee has been considered as a direct indicator of the socket quality fit and comfort. Therefore, researchers have been very interested in quantifying these interface stresses in order to evaluate the extent of any potential damage caused by the socket to the residual limb tissues. During the past 50 years a variety of measurement techniques have been employed in an effort to identify sites of excessive stresses which may lead to skin breakdown, compare stress distributions in various socket designs, and evaluate interface cushioning and suspension systems, among others. The outcomes of such measurement techniques have contributed to improving the design and fitting of transtibial sockets. This article aims to review the operating principles, advantages, and disadvantages of conventional and emerging techniques used for interface stress measurements inside transtibial sockets. It also reviews and discusses the evolution of different socket concepts and interface stress investigations conducted in the past five decades, providing valuable insights into the latest trends in socket designs and the crucial considerations for effective stress measurement tools that lead to a functional prosthetic socket.

## 1. Introduction

The incidence of lower limb amputations is increasing worldwide due to the high rate of traffic accidents and vascular-related diseases [1,2]. Transtibial amputees often use a prosthesis (artificial limb) as a rehabilitation tool to restore their appearance and daily activities [3]. The prosthesis consists of several essential components such as socket, shank, ankle and foot. The socket provides the coupling between the residual limb (stump) and the remaining components of the prosthetic device. The fitting and design of socket is the most challenging procedure due to the uniqueness and complexity of each amputee’s residual limb [4]. Uncomfortable prosthesis due to misfitting at the residual limb-socket interface may lead to excessive stresses, pistoning (vertical movements within the socket), skin irritation and ulcers, and even to potential reamputation [5,6]. Therefore, a smooth load transfer between the residual limb and prosthesis is crucial for a functional socket and the amputee’s satisfaction. This can be achieved by concrete understanding of the interface pressure distribution inside prosthetic sockets as it has a major effect on the socket quality fit and comfort [4].

Over 45 years ago, the interface pressure was not quantitatively assessed [7]. Instead, prosthetists make a clear check socket and then assessed the pressure distribution under static weight-bearing conditions based on the skin color. Skin blanching indicated areas with higher contact pressures than those with normal skin color. For further assessment, experienced prosthetists would insert stick corsets at the residual limb-socket interface to check the socket fit [7,8]. This process requires highly skilled prosthetists/clinicians and does not even provide an overall picture of actual pressure distribution within prosthetic sockets.

Since the late 1960s, researchers and prosthetists have been developing a variety of force transducers in order to map the pressure distribution during/after socket design, thus improving socket fit and comfort [9]. Selecting suitable transducers is challenging and relies on the specific experimental and clinical environment. Few guidelines to be considered when selecting the sensing tool and its mounting technique have been reported [10,11]. The ability of the sensing tool to measure normal and shear stresses is of great importance. Besides, the transducer mounting should be easy and quick. For experiments outside the lab, the sensing system must consume little power to be capable of longer monitoring intervals using batteries. Other requirements include high accuracy and frequency response, enhanced sensitivity, and low hysteresis and drift.

Several measurement techniques have been reported in the literature, including strain gauges, piezoresistive, capacitive, and optical sensors. This article aims to provide a comprehensive overview of the evolution of various transtibial socket concepts as well as the operating principles, advantages, and disadvantages of conventional and emerging techniques used for measuring normal and shear stresses at the interface between the residual limb and prosthetic sockets. It also summarized and discussed the interface stress investigations conducted during the past five decades using the aforementioned techniques. The authors believe that this work will help prosthetists/researchers select the appropriate sensing tool for their future applications.

## 2. The Evolution of Transtibial Socket Designs

The artificial joint-corset transtibial prosthesis that employs a thigh corset, with or without a waist belt as suspension system, had been used many years before Radcliffe introduced the patellar tendon bearing (PTB) sockets in the 1950s [12,13,14]. The PTB socket is commonly made of laminated woven materials together with acrylic resins or of molded thermoplastic sheets [15]. Detailed fabrication procedures can be found in [15,16]. The socket structure provides partial enclosure of the patella tendon (distal third of the patella) and extends the medial and lateral aspects of the socket higher up to the level of adductor tubercle of the femur in order to ensure knee stability and share body weight bearing. The posterior aspect is flared out proximally to allow comfortable knee flexion and prevent excessive pressure on the hamstring tendons. The socket is lined with a cushioning material, made of 5-mm thick polyethylene foam (i.e., Pelite), to reduce the interface pressure between the residual limb and socket [17]. This socket design was widely used with a variety of suspension methods [18,19] to secure the coupling between the residual limb and PTB socket with or without the insertion of a Pelite liner [2]. These suspension methods include suprapatellar cuffs with or without waist belts [12], supracondylar suprapatellar (SCSP) [20], supracondylar (SC) [21], and figure-of-8 suprapatellar straps [22], and rubber sleeves [23].

The PTB design concept distributes loads over the pressure tolerant areas of the residual limb; such as the patellar tendon (PT), the medial flare of the tibia, anterior muscular compartment, and popliteal area, while pressure is relieved on intolerant areas; such as the fibula head, anterior tibia crest, and anterior distal tibia [24,25]. This concept had shown satisfactory results for up to 90% of amputees [26] and still one of the most commonly used socket types [27]. Although PTB socket design provides a good fit, it causes suspension problems and produces concentrated pressures on tolerant areas resulting in skin stretching, which is one of the major causes of residual limb injuries [28,29]. Additionally, the fabrication of PTB sockets is laborious and time consuming and necessitates a skilled prosthetist [16]. It is also not suitable for amputees with sharp bony and/or sensitive residual limbs.

Later in 1986, Kristinsson introduced an alternative socket concept called “Total Surface Bearing (TSB)” sockets with silicone liners [30]. Its shape is significantly different from traditional PTB socket in that it is not indented at the PT and posterior popliteal regions [31]. In addition, the pressure is uniformly distributed all over the residual limb so that no peak pressure occurs. With TSB sockets, residual limb soft tissues are exposed to tolerable compressive pressure while the bony areas are stabilized in the residual limb [4]; thus no damage to skin due to excessive loads occurs when silicone liners are employed [32]. This is because the silicone liner materials are pliable and closely follows the whole contour of the stump surface [24,33]. Two major suspension methods are in use to couple the TSB sockets to the residual limb; either by a single pin attached to the distal end of the silicone liner or through circumferential seals that produce vacuum at the socket [32].

TSB sockets exhibit prominent advantages over PTB sockets such as superior suspension due to the full adhesion of the silicone liner to the residual limb, protection of the residual limb, better cosmetic appearance, and improved function [24]. Generally, amputees have stated preference with prosthetic sockets incorporating silicone liners as suspension systems [34]. In spite of the overall satisfaction with silicone suspension systems, they exhibit numerous disadvantages including perspiration, itching, the increase of bulk around the knee and proximal circumference of the socket, volume changes of the residual limb during daily activities, difficulties in donning and doffing, and milking phenomenon. Recently, new suspension systems (air pneumatic [35] and magnetic systems [36]) have been proposed, and it is claimed that they can overcome the drawbacks of commonly used systems with regard to the volume changes over time, donning and doffing, and the pain induced by the milking phenomenon.

Rapid Prototyping (RP) technologies have been recently investigated as an alternative candidate to the traditional sockets manufacturing techniques [37,38]. The acquisition of the residual limb shape is done through a 3D laser scanner that produces digitizing data. CAD/CAM software is then used to convert the digital information into a 3D shape of the residual limb. The shape is saved in a suitable format for rapid prototyping. It is believed that in addition to reducing the socket fabrication time from days to hours, which undoubtedly favorably affects the cost, it eliminates the tedious steps of traditional socket fabrication such as making the negative and positive molds, rectification, and finishing. However, a feasibility study by Tan et al. found that RP socket functional characteristics were very similar to that of traditional sockets in terms of pressure distribution and the usual drawbacks [15,37]. In 2006, Faustini and colleagues modified the RP-fabricated socket with integrated compliant features (thin-walled sections) to provide high contact pressure relief at the interface between the residual limb and socket [39]. Rogers et al. have assessed the performance of RP sockets with the variably compliant features and found that it obviously reduced the pressures on sensitive areas of the residual limb which led to better comfort and fit of the prosthesis, suggesting that evaluation of long-term durability of compliant sockets with a large number of subjects would provide better insight into the practicality of this design concept [40]. Later in 2013, Sengeh and Herr from the Media Lab at the Massachusetts Institute of Technology (MIT) introduced and evaluated a 3D printed variable-impedance prosthetic socket with compliant features aiming to lower the interface pressure over bony protuberances [41]. In this design, the smoothly varying socket wall impedance is inversely proportional to the impedance of the underlying anatomy of the residual limb. This design reduced the contact pressures when compared with rigid conventional socket and a 16% increase in the self-selected walking speed of the participant was observed. However, the socket was nearly three times heavier than the conventional carbon socket due to the poor mechanical properties of the 3D printed materials and the large socket-wall thicknesses necessary to achieve structural integrity. A broader research to reduce the socket weight with maintaining its structural integrity is needed. Moreover, clinical studies that include a large number of subjects is necessary to better understand the relationship between excessive socket pressure and socket variable impedance properties using widely used interface pressure measurement techniques.

## 3. Transducer Mounting Techniques

**Figure 1:**
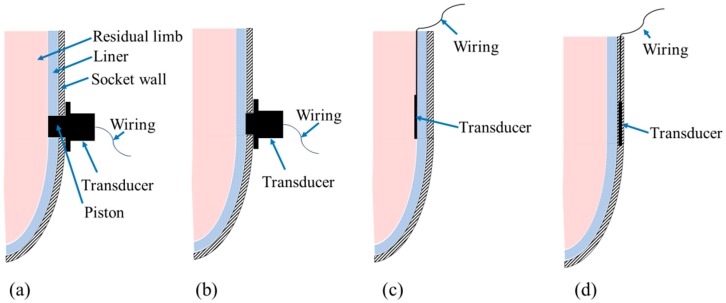
Transducer mounting techniques: (**a**) transducer mounted on socket wall through drilled hole and the piston extended to be in direct contact with residual limb skin; (**b**) the same mounting technique with a slight difference that the piston is flush with the inner socket face and does not penetrate the liner; (**c**) transducer inserted inside prosthetic socket; and (**d**) transducer embedded in the socket wall.

Based on the literature, interface stress measurement transducers can be mounted using different techniques: transducers mounted on socket wall, transducers inserted in socket, and transducers embedded in the socket wall (see Figure 1).

### 3.1. Transducers Mounted on Socket Wall

This technique was first used in 1968 by Appoldt et al. to study pressures in transfemoral sockets [42]. The strain gauge (SG) is the most common sensing element employed for this mounting technique. An assembly of SG sensing elements and cylindrical piston are configured in a cylinder-like housing mounted onto the socket wall in locations of significance through drilled holes, as shown in Figure 1a,b. The piston must be flush with and normal to the socket inner face in order to transfer the stresses on the residual limb to the SG sensing elements.

Many investigators have used this mounting technique during the past five decades to evaluate the static and dynamic interface pressure profiles within transtibial sockets [43,44,45,46]. SG-based transducers are undoubtedly able to measure both normal and shear stresses with high accuracy and sensitivity, however, placement of these transducers requires a modified check socket with holes which is laborious and may alter the residual limb-socket interface pressure distribution. Moreover, the bulkiness of transducers increases the weight of the prosthesis during the experiments, affecting the accuracy of interface pressure measurements. It also dismisses the pressures at the areas in between the transducer sites, offering lower spatial resolution [8]. These limitations impeded the use of this technique in clinical settings.

To overcome these shortcomings, Sewell et al. have recently designed and clinically tested an artificial intelligence approach, particularly inverse problem analysis. This approach could predict the static and dynamic pressures inside transtibial sockets from strain data collected by SGs attached directly to the socket outer surface. No holes through the socket wall are required to accommodate the SG transducers [47,48,49]. The Artificial Neural Network (ANN) approach can find a complex non-linear transfer function that relates the surface strain to the applied internal pressures. To find this relationship, a loading device for applying known pressures inside the socket is used and the SGs collect the related surface strain data that will then be stored as ANN inputs and outputs pairs. A large number of these pairs were required for the ANN to accurately train (i.e., find an accurate relationship between the inputs and outputs). This approach might allow the prosthetists to quantitatively analyze the interface pressures within prosthetic sockets in clinical settings. In addition, these SG transducers can be mounted easily and rapidly. However, only one subject has been involved in the experimental validation and the results need to be compared with other commercially available techniques such as F-Socket sensing mats (Tekscan, Boston, MA, USA).

### 3.2. Transducers Inserted in Socket

This technique does not require a modified check socket with holes as the transducers are quite thin and can be inserted between the residual limb and liner or between the liner and prosthetic socket [2], as shown in Figure 1c. SG, piezoresistive, capacitive, and optical-based transducers have been inserted in sockets. Sonck et al. was the first to assess pressures in transtibial sockets by inserting a diaphragm deflection SG-based transducer in transtibial sockets [50]. F-Socket sensing mats have been introduced later, providing higher spatial resolution. Several F-Socket transducers are usually used simultaneously to map pressure distribution all over the residual limb aspects. However, these sensing mats exhibited hysteresis and drift. Furthermore, the F-Socket sensels might crease and fail, affecting the measurement accuracy.

### 3.3. Transducers Embedded in Socket Wall

This mounting technique is being developed by researchers from Centre for Applied Biomechanics (CAB) at the University of Malaya, Malaysia. The researchers have conducted several attempts to embed Fiber Bragg Grating (FBG) sensors in the socket wall during socket fabrication (see Figure 1d). The FBG sensors are sandwiched in between stockinet layers before injecting the resin material all around the positive mold of the residual limb. Due to their high sensitivity to dynamic loads [51], the internal strains induced within the socket wall during amputee ambulation can be translated into interface pressure values using the inverse problem analysis approach. FBGs are very small sized [52] and can be multiplexed to enable a larger sensitive area [53], thus they provides higher resolution than some other reported transducers. It is hypothesized that the FBGs with this configuration can also be used to assess the mechanical performance of socket wall materials during ambulation, running and cycling.

## 4. Types of Transducers

A variety of interface stress measurement methods have been used within transtibial sockets including SGs, piezoresistive, capacitive, optoelectronic, and optical fiber transducers. Table 1 summarizes their structure, mounting techniques, advantages, and disadvantages. Detailed background, working principles, and applications of such systems in transtibial sockets are highlighted in the following sub-sections.

### 4.1. Strain Gauge-Based Transducers

**Figure 2:**
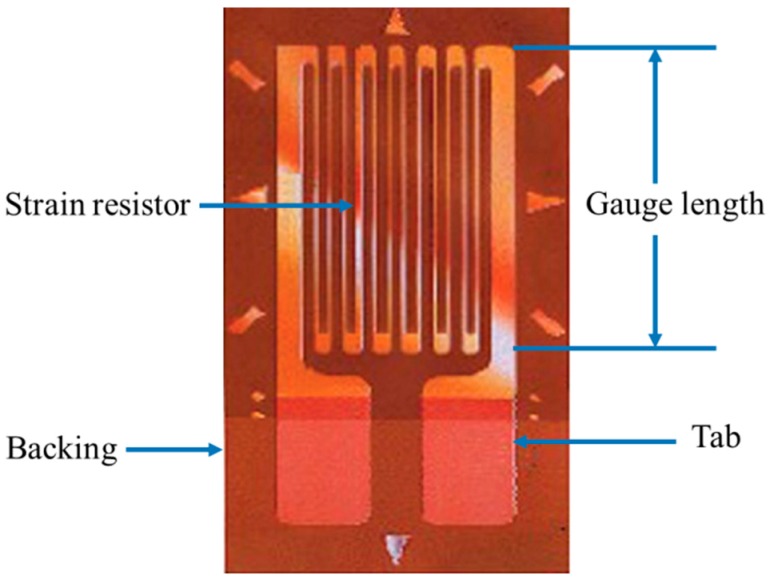
Traditional strain gauge [78].

SGs are small patches of silicone or metal that exhibit a change in their electrical resistance in response to any applied mechanical strains (see Figure 2) [54,55]. They are very sensitive and susceptible to humidity and heat changes; therefore, they are very often used in Wheatstone bridge configurations to overcome these problems [56]. The relative change of resistance (ΔR/R) with respect to that change in strain (ε) determines the gauge factor (GF) as in the equation below:

(1) $$GF\;=\;\frac{\Delta R/R}{\varepsilon }$$

SG-based transducers have been widely used in many applications [54]. Their use in lower limb prosthesis for pressure measurements established in late 1960s [42]. They have been used either as diaphragm deflection transducers inserted in socket to measure only pressure [57] or as a piston-type transducers mounted on the socket wall to measure both normal and shear stresses [58]. The first reported diaphragm deflection SG-based transducer capable of measuring only direct pressure was Kulite sensor in 1970 [50,59]. Its sensing element diameter and thickness are 3.2 and 0.8 mm, respectively. It is monolithic and has a stiff backing to keep it flat when attached to curved surfaces. In spite of its simplicity, high sensitivity and lightweight, it exhibited several limitations. Its stiff backing mismatches with the residual limb tissues and liner, causing stress concentrations at the sensor edges especially at the anatomically curved areas, putting the skin in local tension and altering the pressure distribution [59].

Another limitation is that it measures loads at isolated sites, dismissing the stresses between the measurement sites. Rae and Cockrell attempted to overcome the latter problem by taping several Kulite sensors together in a grid-like array to achieve stress measurements within a certain area [57]. However, the array was subjected to crosstalk due to its rigidity. In addition, the group of cables restricted the subject movement and altered the amputee’s normal gait.

To lessen the above limitations such as the crosstalk and edge stress concentrations, the piston-type transducer mounted on the socket wall has been introduced. Holes are drilled in the socket wall to affix the piston-housing cylinder at sites of clinical interest with the aim to place the transducers flush with the residual limb-socket interface. Appoldt and Bennett were the first to come up with this transducer concept [42]. The pressure transducer concept was a cylinder-housed plunger piston-type force gauge. The piston transfers the interface pressure to a small beam equipped with a SG and clamped at its ends to the distal end of the cylinder frame. This transducer showed appropriate sensitivity and was insensitive to crosstalk. However, it was a unidirectional transducer measuring only direct pressures.

The external stresses acting on a residual limb are a combination of normal and shear stresses. Thus, shear stresses are no less important than direct pressures as they might reduce skin blood flow, producing lesions in skin [10]. Consequently, Appoldt et al. introduced in 1970 a shear stress transducer that is able to fit in the same holes used for direct pressure measurements and be flush with the interface [59]. Later, Sanders and Daly introduced the first in-socket-wall transducer that is capable of measuring stresses in three orthogonal dimensions simultaneously with exceptional advancements [45]. Thereafter, several researchers improved the SG-based in-socket-wall transducer designs which contributed to better understanding of interface stresses in transtibial prosthetic sockets [11,16,79,80]. Clinical investigations of normal and shear stresses that involve a number of amputees were then conducted during standing and walking [44,80,81,82,83].

Despite the advances in SG-based transducer designs, it is still used as a research tool rather than as a clinical device as the socket to which they attach must be permanently modified with holes drilled through the socket wall [7]. Furthermore, the bulky size of piston-type SG transducers and their data cables increased the prosthesis weight, distorting the stress measurement [10]. Moreover, SG based transducer experiments are usually conducted in lab since they require a relatively more power to operate. For outside of the lab, they operate for short-term use in research settings using batteries [11,84]. Other sensing technologies, such as piezoresistive and capacitive, requires less power and thus may be applicable for longer monitoring intervals [11].

### 4.2. Piezoresistive Transducers

Most researchers and instrument builders prefer to use piezoresistive sensors such as Force Sensing Resistors (FSRs) [85] due to their distinguished features including thin construction, small profile, flexibility, good sensitivity, relatively simple structure, and ease of use [60,61]. FSRs can be made in various shapes and sizes so as to be utilized for many applications to measure rate of changes in applied forces and detect contact pressure between two surfaces. In contrast to SGs that could be either positioned within prosthetic sockets or mounted on socket wall, all piezoresistive sensors are very thin sheets; ideal to be positioned in-situ inside the prosthetic socket.

Standard FSRs are made of a pressure sensitive element in the form of an elastomer, conductive ink, and conductive rubber or carbon fiber that is sandwiched between two layers of flexible polyester films glued by an adhesive to form a piezoresistive pressure sensor [54]. Typically, they work as a variable resistance with a magnitude larger than 1 MΩ when unloaded. If an increased pressure is applied onto the surface of the sensor, the resistance drops accordingly [60,86]. The electrical resistance is calculated using the following formula:

(2) $$R\;=\;\frac{\rho \;\times \;l}{A}$$

where ρ, *l*, and *A* denote the bulk resistivity, length, cross-sectional area of the piezoresistor respectively. Usually, the change of resistance is converted into corresponding voltage output using the Wheatstone bridge configuration [87,88].

**Figure 3:**
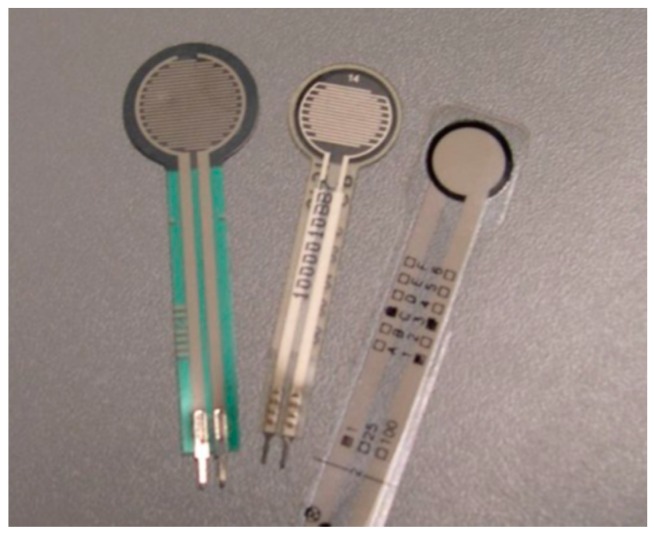
Three common types of FSRs: Interlink, LuSense, and FlexiForce [85].

Several FSRs are commercially available, such as the Interlink FSR (Interlink Electronics, Inc., Camarillo, CA, USA), the LuSense PS3 (LuSense, Luxembourg), and Tekscan FlexiForce A201 (Tekscan, Boston, MA, USA) shown in Figure 3. Some researchers have experimentally investigated the differences between these different designs in terms of linearity, dynamic accuracy, time drift, and repeatability [60,85,89]. The FlexiForce performed better when large slowly-varying forces are applied infrequently for long durations. It also showed the highest precision, but with higher noise than the other two. Unfortunately, these piezoresistors can measure contact forces at only one site while it is required to use multiple strips or an array of piezoresistors to cover larger areas on the residual limb. Zhang and his colleague employed parallel resistive strips to allow pressure measurement at many contact points [90]. Ruda et al. have recently attempted to make a sensor network containing five FlexiForce sensors aligned in a special configuration and embedded in a flexible, thin sheet of acetate [68]. Unfortunately this configuration is still inefficient to measure residual limb stresses since it has a very small sensing surface.

**Figure 4:**
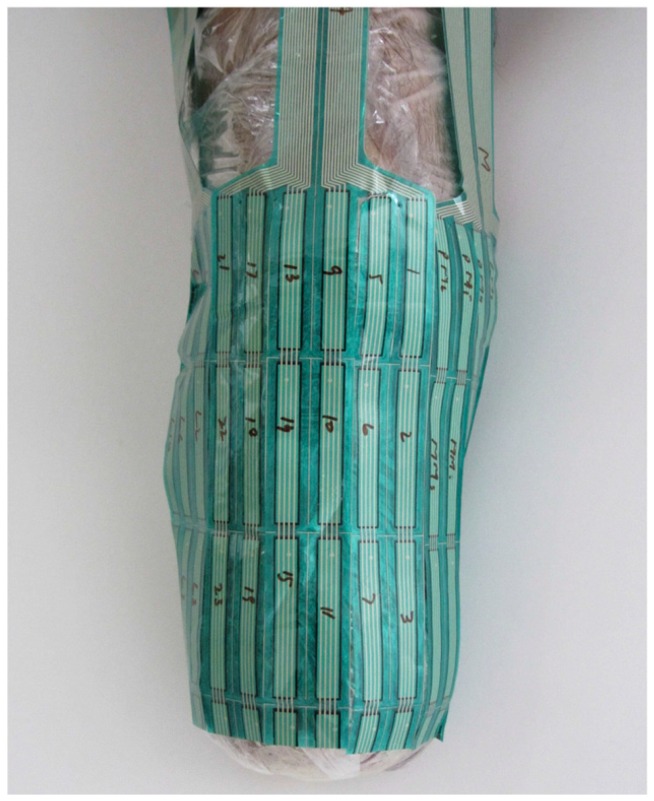
Four F-socket transducers covering all aspects of the residual limb to give an overall impression of pressure distribution inside transtibial prosthetic sockets [92].

The Rincoe Socket Fitting (RG Rincoe and Associates, Golden, CO, USA) and F-Socket (Tekscan,) systems are the most commonly used piezoresistive sensing sheets for interface pressure measurement inside prosthetic sockets. Both systems require no modifications in sockets, making them superior over piston-type SGs. The Rincoe Socket Fitting System is a combination of 60 FSRs embedded in six polyvinilidyne fluoride strips with a thickness of 0.36 mm. Each strip is composed of 10 sensing points [91]. They are factory calibrated, but a calibration table is provided by the manufacturer. The F-Socket transducer is also based on FSR technology. It is constructed of 96 individual sensing points (sensels) arranged in a matrix of 16 rows and six columns [60], as shown in Figure 4. It therefore provides higher spatial resolution of the pressure distribution than Rincoe Socket strips do. Each sensel performs as a variable resistance that changes its value upon the application of pressure. F-Socket transducer system does not require sophisticated electronics. Prior to each clinical trial, F-Socket mat however needs to be equilibrated and calibrated, according to the manufacturer’s instructions [62].

To the best of the authors’ knowledge, Engsberg et al. was the first to investigate pressures inside transtibial sockets using the F-socket system [63]. Two transtibial amputees participated in this study and the pressure distribution was assessed in static and dynamic conditions. Houston et al. later included more subjects (four amputees) to evaluate the F-Socket transducers [64]. These two studies found that the pressure values measured with the F-Socket system were similar to those previously reported with other types of pressure measurement systems [9,45]. These results were optimistic about the potential use of F-Socket system in clinical settings. To date, neither the manufacturer nor these two prosthetic investigations have discussed the calibration of F-socket transducers, their accuracy, reliability or other issues pertaining to their use [7,8,9].

Later, several researchers have assessed the validity and reliability of F-Socket transducers for prosthetic use in a clinical environment [65,66,67]. Buis and Convery investigated the encountered calibration problems and the possible techniques to reduce inaccuracy in clinical measurements [65]. Hachisuka et al. studied the performance of Tekscan pressure transducers when subjected to rapid and repetitive movements [66]. Despite the sensor drift, hysteresis, and temperature sensitivity, the sensor reproducibility and sensitivity was considered satisfactory under laboratory conditions. Polliack and colleagues have compared the Rincoe Socket Fitting and F-Socket systems in terms of reliability, accuracy, hysteresis and clinical validity when these sensors were subjected to tests in flatbed and custom-designed pressure vessels [9]. The findings indicated favorable results for the F-Socket pressure sensing mat (8% (flatbed) and 11% (mould)) compared to the Rincoe Socket system (25% (flatbed) and 33% (mould)) with respect to its accuracy errors only. The hysteresis and drift tests showed better results with the Rincoe system. Generally, they believed that both systems can be used in clinical settings for residual limb-socket interface pressure measurements. Since then, the F-Socket system has been the most commonly used pressure sensor for prosthetic sockets. As above-mentioned, the main drawback of F-Socket sensors is related to their disability to measure shear stresses and their hysteresis that causes low frequency response when compared with capacitive sensors [54,88].

### 4.3. Capacitive Transducers

A capacitance sensor consists of a dielectric material sandwiched between two parallel conductive surfaces. It could be configured based on two displacement principles; the first approach depends on the change in the overlapping surface area between the two conductive surfaces which makes it more attractive due to its high precision. The second approach depends on the distance between the two conductive surfaces [54]. The capacitance can be expressed as:

(3) $$C\;=\;\frac{A\;\varepsilon_{0}\varepsilon_{r}}{d}$$

where C is the capacitance, *A* is the overlapping area of the two surfaces, *ε*_0_ is the permittivity of free space, *ε*_r_ is the relative permittivity of the dielectric material and d is distance between the plates [68].

Capacitive sensors have also been used in prosthetic applications [69,70,71,72] and could be mounted inside and/or outside transtibial sockets. Meier et al. reported in 1973 the first attempt to use a capacitive sensor for pressure measurements in prosthetic sockets [73]. They developed flexible, inexpensive capacitive sensors that were each 2-mm thick and could be inserted at the interface between the residual limb and socket. Based on bench tests performed, the operational accuracy was ±20%. In 1998, Polliack and his colleagues contacted Novel Electronics Inc. (Saint Paul, MN, USA), requesting a capacitive-based sensor design, similar to the one that had been commercialized by the company for seating assessments (i.e., wheelchair), that is capable of measuring interface pressures inside prosthetic sockets [67]. The “Novel” prototype capacitive sensor was then designed for their study as a matrix array of 16 sensing sites (4 × 4) mounted in silicone substrate (2.5 cm × 2.5 cm) with a thickness of 0.63 mm. It is usually inserted between the skin and liner or between the liner and socket. Bench tests using compressed air were performed to assess the validity of the “Novel” prototype sensor in terms of accuracy, hysteresis, effect of curvature, and drift responses in both a flatbed chamber and a custom modified pressure vessel. Novel sensors were then positioned at nine different locations on the residual limb positive mold enveloped with a silicone liner. For clinical evaluation of this sensor design, two transtibial amputees participated in this study. The results were encouraging. The bench tests and clinical study showed no noticeable sensor drift occurred between pre- and post-test calibration values after three hours of continual use. It also showed acceptable reliability and accuracy [69]. These findings made capacitance sensors much superior to piezoresistive sensors [93,94]. Novel Electronics, Inc has since then commercialized this capacitance socket sensor. However, this sensor design is still unidirectional, measuring only direct pressures. Later, several tri-axial stress sensors have been reported, but their use in prosthetic sockets was limited due to their rigid substrates that do not comply with the residual limb geometry [58,95] and their sophisticated manufacturing techniques that hinder low cost fabrication of multiple sensor arrays [74].

3D printing technology offers a low-cost and versatile solution with capability of adopting irregular shapes which represents a key advantage for potential applications at residual limb–socket interfaces. In 2015, Laszczak and his colleagues developed and validated a capacitive-based 3-D printed stress sensor that is small in size and could simultaneously measure normal and shear stresses inside prosthetic sockets [1]. It is made of a flexible frame (20 mm × 20 mm), with a thickness of 4 mm. Firstly, it was analyzed using FEA and then evaluated using lab tests. The results showed that the sensor is capable of monitoring both pressure and shear at stresses up to 350 kPa and 80 kPa, respectively. These promising results suggest that these sensors could have a strong potential for effective pressure and shear measurements at the critical residual limb-socket interfaces.

Although capacitive sensors require more sophisticated electronics, they are found to provide higher sensitivity and flexibility, lower temperature dependency, more robust structure, lower power consumption, better frequency response and a larger dynamic range than piezoresistive devices. The main drawback is their susceptibility to crosstalk noise, especially when arranged in a mesh configuration, and therefore require relatively sophisticated electronics to filter out this noise [54,69,88].

### 4.4. Optical Sensors

Fiber optical sensors (FOS) were introduced to the field of medicine in the 1960s for cardiac, endoscopic, and intravascular applications [96]. In recent years, FOS have demonstrated an extensive and rapid growth in many basic life sciences research and medical applications [97,98,99,100,101,102] as a useful sensing device for measuring strain [103,104], pressure [105], force [106], temperature [107] or refractive index [108]. The most common sensing techniques applied to FOS in the field of biomechanics are based on intensity [109], phase [110], and wavelength modulation [111], the latter being associated with the operation of fiber Bragg grating sensors (FBGs). FBGs have attracted many researchers due to their superior advantages, including high sensitivity, durability, immunity to electromagnetic interference (EMI), mutiplexability and resistant to harsh environments [112,113]. In biomechanics and rehabilitation engineering, FBGs have been demonstrated for measurement of a wide variety of parameters; including strain inside and on the surface of intact and plated bones, shrinkage stresses in bone cement during polymerization, pressure mapping in orthopedic joints, stresses in Intervertebral discs, deformation in chest wall to study lung biomechanics, pressure distribution in human-machine interfaces, forces induced by tendons and ligaments, angles between body segments during gait, and many others in dental biomechanics [75]. Indeed, they seem to be the potential alternative to those SGs, piezoresistive, capacitive, and electromechanical pressure sensors [55,114].

**Figure 5:**
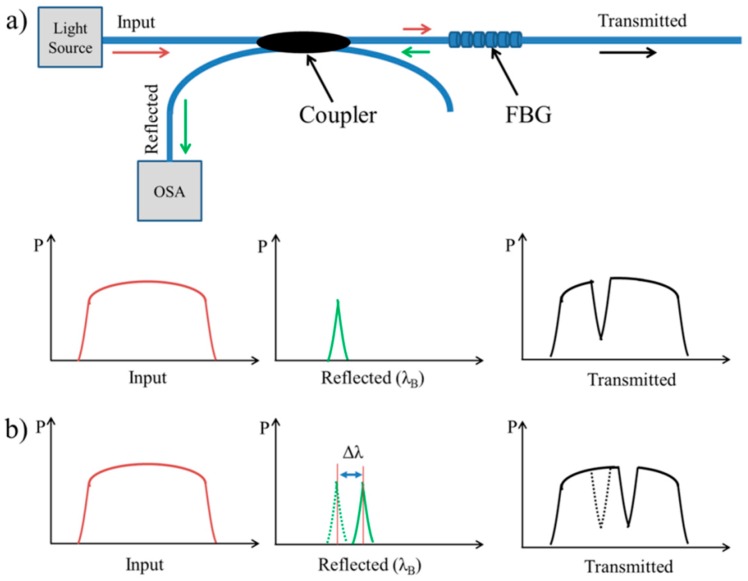
FBG sensor working principles; (**a**) the light spectrum (brown color) passes through the FBG fiber and a narrow wavelength band (green arrow) is back-reflected and monitored by OSA; (**b**) the back-reflected wavelength is shifted (Δλ) shortly after applying external perturbations [75].

The FBG has a longitudinal periodic variation of the refractive index n_eff_ written in the core of optical fiber in different methods; phase mask, direct writing, or a combination of phase mask and interferometry, for generating the required spatial pattern [115,116]. When an optical fiber with an FBG is coupled to a light source and subjected to any external mechanical forces, the light passing through it will be back-reflected by the FBG itself at a Bragg wavelength, λ_B_, depending on the spacing between the periodic variations and the strain-optic effect [117]. The working principles are illustrated in Figure 5. The reflected spectrum depends on the refraction index (n_eff_) and the Bragg grating period (Λ) of the grating, according to the following equation:

(4) $$\lambda_{B}\;=\;2n_{eff}\;\Lambda$$

In the last decade, a few studies have demonstrated the feasibility of FBG sensors in lower limb prosthetic sockets. The IASiS project was launched in 2008, aiming to use FBG sensors in rehabilitation engineering such as prosthetic sockets [19]. Kanellos and his colleagues developed a flexible 2D FBG pressure sensing pad that could be suitable for pressure mapping in the residual limb-socket interfaces [118]. They also studied the reliability and durability of this sensing pad in prosthetic socket applications through the identification of three basic fabrication parameters: fiber embodiment in the polymeric pad, pad thickness, the type of FBG fiber [119]. They found that the optimum sensing pad that guarantees enhanced durability and required sensitivity is when the FBG fiber is embedded at the center of 3-mm thick PDMS polymeric sensing pad. Later in 2015, Al-Fakih et al. conducted a similar study to assess different embedding materials when the sensing pads are attached to Pelite and silicone liners [3]. It was obvious that the FBGs embedded in harder materials exhibit higher sensitivity and accuracy with both types of liners. Tsiokos et al. suggested a pressure management system that works in two steps [120]. The first step is to measure pressure loads across the residual limb-socket interface in order to generate pressure maps that define pressure distribution. The data obtained from the sensors drive the pressure actuators attached to the inner socket wall to redistribute the loads throughout the interface for pressure relief. However, there are no subsequent studies found in the literature to validate this distinctive system.

The capability of FBG sensors to measure interface pressures between the residual limb and socket in-situ was first investigated by researchers from the University of Malaya Center for Applied Biomechanics (CAB) in 2013 [121]. A pressure sensor was formed by embedding a single FBG at the neutral layer of a polymeric sensing pad and attached to the PT region of the socket. A heavy duty balloon was positioned in socket and inflated/deflated in a cyclic pattern using air compressor to mimic the actual amputee’s gait. The sensor showed good reliability and acceptable hysteresis, opening up the evolutionary road into smart FBG-based socket pressure monitoring systems. Later in 2015, the same researchers improved the sensor design and fabricated an expandable array of FBGs that could be inserted within prosthetic sockets to provide an overall impression of the pressure distribution throughout the interface [3]. Only one subject took part in this study to clinically validate the findings of this sensing pad. The results showed that the FBG array is capable of successfully measuring the interface pressure within the prosthetic socket. To assess the validity of the FBG sensor, its results were compared to those obtained using the commercially available F-socket transducers. FBG sensors produced higher pressure magnitudes at all the sensing sites. This might be due to the thickness of the FBG sensing pads, yet the pressure pattern was similar for both sensor types. This sensor design needs to be tested on a larger sample size to further validate the findings.

**Figure 6:**
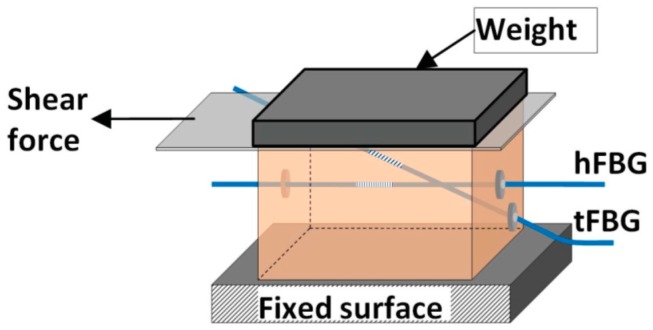
Diagram of normal and shear stress sensor using PFBGs [122].

Monitoring shear stresses is increasingly important in prosthetic sockets. All previous FBG sensor designs were capable of measuring only direct pressures. Zhang et al. reported a polymer FBG (PFBG) sensor capable of measuring normal and shear stresses [122]. The sensor consisted of two PFBGs embedded in a soft PDMS matrix, one of which is horizontally placed while the other is tilted across the matrix as shown in Figure 6. Four gaskets were used to fixate the gratings at their ends. The matrix was experimentally demonstrated by simultaneously applying normal and shear stresses to induce strains on FBGs. The results showed that the sensor performed well and could determine the pressure and shear stresses. In spite of its high sensitivity, this soft sensor could be suitable for making low normal and shear stresses measurement. Further studies are needed to study the sensor repeatability and durability before being used for practical applications.

**Figure 7:**
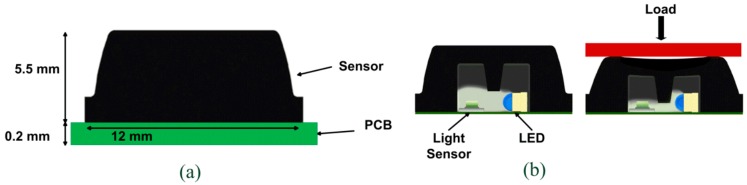
Optoelectronic pressure sensor for prosthetic applications [124].

Opto-electronic sensors that incorporate a printed circuit board (PCB) have been utilized in prosthetic research for pressure and shear measurements. An optoelectronic pressure sensor made of an external silicone bulk structure and a printed circuit board which accommodates an array of sensitive elements has been introduced to monitor pressure distribution at human-machine interfaces such as prosthetic sockets and the foot-ground interface [76,123,124]. Each sensitive element is composed of a light transmitter, a LED and a receiver, a photodiode. Figure 7 shows the sensor structure and how the silicone bulk plays a major role in the transduction principle. When pressure is applied onto the sensor, the silicone bulk deforms itself. The light intensity received by the photodiode varies proportionally its output voltage. However, this design was not practically evaluated in prosthetic sockets.

A different configuration of optoelectronic sensors was developed by Missinne et al. in 2010 [125]. They demonstrated a new shear sensor concept that is very thin and flexible and works based on the changing of optical power between the emitter (laser) and receiver (photodiode) separated by a deformable sensing layer of PDMS. The sensor structure deforms when subjected to external shear forces, resulting in varying the power intensity received by the photodiode. This sensor concept was found to be reproducible and limitedly influenced by normal stresses. Later in 2012, a group of researchers from Sandia National Labs in Albuquerque, NM, USA, described the development of an inexpensive three-axis optoelectronic sensor that is composed of small, surface-mount integrated circuits with multiple layers of silicone elastomer [77]. The sensor was able to make normal and shear measurements. The integrated circuit contains five small packages of a LED and phototransistor; one to detect normal loads, two to detect shear in one direction, and two others to detect shear in the orthogonal direction. The top layer of the elastomer was constructed of a square reflective silicone centered over the sensor for detecting normal loads and an absorptive opaque silicone placed at the boundaries. When normal loads are applied, the reflective silicone moves the LED (emitter) into the phototransistor (detector), causing more reflected lights into the detector. Shear loads are detected by the changing ratio of absorptive to reflective materials between the emitter and the detector. The sensor exhibited modest drift and hysteresis. Some media news reported that a number of these sensors had been embedded in silicone liners to measure normal and shear stresses at the residual limb-prosthetic socket of a number of transtibial amputees [126], but no published work demonstrating this sensor liner can be found in the literature.

The optical sensors, however, have a few disadvantages such that their full operation might be hampered due to any damage to the optical fiber or optoelectronic components [112]. In addition, electrical type systems such as optoelectronic sensors are susceptible to EMIs [127]. Contrastingly, the key advantage of FBG sensors is their immunity to EMIs, making them the potential candidate to replace the existing measurement systems being used for transtibial prosthetic socket pressure and shear measurements.

## 5. Prosthetic Interface Stress Measurement in Different Socket Designs

Many studies have been conducted using the aforementioned force transducers to make the normal and shear measurements for amputees wearing PTB and TSB sockets during level walking, running, stairs, and slops. These studies are grouped based on the socket design concept. Table 2 also summarizes the applications of various pressure transducers based on their transduction methods.

### 5.1. PTB Sockets

Rae and Cockrell used linear and area arrays of diaphragm SG-based pressure transducers (Kulite transducers) inserted in PTB sockets to determine the interface pressure during normal walking at three different conditions: no liner, Kemblo liner, and silicone gel liner [57]. The average peak pressures were found to be the lowest with silicone liners. Pearson et al. also used the same transducers to measure interface pressures in the PTB critical regions for static stance-support positions (prosthetic limb suspended, the weight on both limbs, and the weight fully borne by the prosthesis) and during walking [141]. The results showed that the static pressure when the weight fully borne by the prosthesis with contracted residual limb muscles approaches that of the maximum pressure during walking, which might be used as a clinical measure to provide protection against excessive dynamic pressures. In 1975, the same researchers investigated the effect of rubber suspension sleeve on the suction pressure at the small space between the distal area of the residual limb and transtibial PTB socket during the swing phase. The suspension sleeve played a major role in improving the overall comfort and function of the prosthesis when compared with suprapatellar cuff or thigh band suspension methods [23].

Meier and colleagues employed capacitive sensors to record pressures at five sites on the residual limbs of eight transtibial amputees [73]. The subjects have undergone several clinical trials and the results showed a range of interface pressures up to 415 kPa. Dou et al. have used portable, real-time (50 Hz), telemetric Pliance capacitive sensors that were placed between a 6-mm thick silicone liner and PTB socket to measure pressures at five sites of interest on the residual limb of only one transtibial amputee during walking on stairs, flat, and non-flat roads [6]. However, multiple subjects are required to ensure the effectiveness of such sensors with different liners and socket designs. Unfortunately, the Novel capacitive socket sensor can measure only interface pressures [127], but not shear stresses which are of great interest to researchers and play a major role in skin problems.

Piston-type SG transducers mounted on socket wall were also employed. Their capability of measuring normal and shear stresses is their key advantage. Sanders and colleagues were the first to have conducted a series of studies using piston-type SG transducers to investigate interface normal and shear stresses simultaneously in transtibial amputees using PTB sockets [45,129,142]. They also reported the characteristics of interface stress wave-form shapes and discussed the effects of those characteristics on residual limb tissue mechanics [130]. Later in 1997, they expanded their investigative studies by measuring interface stresses at 13 sites on the residual limbs of amputees wearing PTB sockets [131]. In addition to characterizing the similarities and differences of interface stress wave-form shape among different sites, they investigated the magnitudes of maximal stance phase pressure, maximal shear stress, shear angle, and changes in pressures for each site. Zachariah and Sanders used the same transducers on two male amputees wearing PTB sockets with no liners to address whether the interface stress ratios of standing to walking are the same in different regions of the residual limb [43]. In addition, they questioned the linear increase of interface stress magnitudes as the amputee progresses from minimal weight-bearing to equal weight bearing, to full weight-bearing, to the stance phase of walking. They found that standing stresses were moderately predictive of peak walking stresses only with full weight-bearing. These findings are in agreement with those obtained by Pearson et al. using Kulite sensors [128]. Non-linearity from minimal to equal to full weight bearing at the anterior region peak pressures was evident.

**Figure 8:**
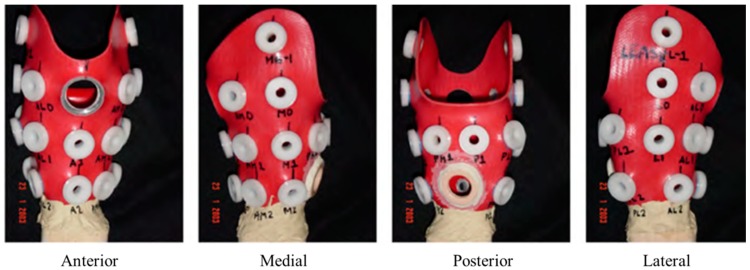
Locations of SG-based transducers on PTB socket [16].

Abu Osman and co-workers have positioned SG-based transducers at 16 sites (see Figure 8), more than in previously reported studies, at all the interesting points, including those located on the high-curvature regions [79]. They investigated to what extent the changing indentation depth at the PT region would affect the pattern of interface pressure distribution and then assessed the correlation that may exist between the pressure magnitudes at PT bar and other sites within the socket [16]. Ten male subjects participated in this study and the PT bar of their sockets was indented inward at 2-mm each, but not more than 4 mm from the original position. The results revealed that altering the indentation depth at PT bar had no effect on the pressure distribution at all sites within the socket, and the subjects who participated in this study experienced no pain or discomfort from removing the PT bar, concluding that the PT bar could be eliminated during the socket fabrication.

### 5.2. TSB Sockets

Goh et al. have also used piston-type SG transducers to investigate the pressure distribution of five amputees wearing TSB sockets fabricated using the pressure casting (PCast) technique [44]. The transducers were mounted on the socket wall at discrete points, and pressure and gait parameters were measured simultaneously during standing and walking. PCast technique was found to be able to produce comfortably fitting sockets. This study showed that hydrostatic method might be an attractive concept as it distributes the pressure evenly across the interface. In addition, it improves socket delivery time. However, the authors did not consider shear stresses.

The pressures associated with pin suspension system used with TSB sockets were first investigated and compared with those obtained from the suction suspension by Beil et al. using five FSRs [143]. The FSR sensors were placed at discrete points on the residual limb for pressure measurements, and an air pressure sensor was used to measure the negative pressure at the distal end of the limb. Eight unilateral amputees were provided with TSB sockets with urethane liners. The results confirmed the hypothesis that during swing phase, higher pressures at the proximal region of residual limb and a large vacuum at the distal end were observed with pin suspension. The researchers concluded that this combination of pressures observed in amputees using pin/lock liners might be the cause of the daily skin problems.

### 5.3. PTB vs. TSB Sockets

Goh et al. compared the pressure distribution of a PCast socket with traditional PTB sockets using piston-type SG transducers [132]. The transducers were mounted at 16 sites of interest within the sockets of four transtibial amputees who volunteered for this study. Two subjects had a similar pressure profile for the two types of sockets. The third subject exhibited a ring of high pressure at the proximal part in PTB socket while the fourth had higher pressure distally at the PCast socket. It seems that due to the low number of amputees participating in this study, the researchers could not determine which socket concept was the best. However, they avoided the high curvature regions such as tibia1 crest, patellar tendon, and fibular head.

Using a limited number of SG transducers (i.e., 16 sites) in previous investigations could not provide an overall impression of the residual limb-socket interface pressure distribution during amputee gait. This is because the stresses in areas between the measurement sites are dismissed. In research and clinical settings, researchers preferred the use of F-Socket system as it could cover more than 90 percent of the interior surface of a transtibial sockets as shown in Figure 4 above. Convery and Buis were the first to use a F-Socket system for comparing the residual limb-socket interface pressure distributions in PTB and Hydrocast (TSB) sockets under dynamic conditions [133,134]. The sensors were attached to the inner socket wall and data were collected during the gait stance phase of a single amputee. The same experimental procedures with a TSB socket were repeated for comparison purposes. Pressure gradients within the Hydrocast socket during gait were found to be less pronounced and more evenly distributed than in PTB sockets with emphasizing that more distal pressure in TSB sockets was noted. In addition, a ring of high pressures was observed at the proximal level of PTB socket with no major distal end pressure.

Dumbleton et al. conducted a similar study, comparing the dynamic interface pressure distribution and patient satisfaction between PTB sockets with Pelite liner and Hydrostatic sockets with silicone liners at various regions on the residual limb during ambulation [135]. A larger number of amputees (*n* = 48) were involved in this study; half wore PTB sockets while the other half wore pressure-cast sockets. The transducers were inserted in between the liner and socket, calibrated using specially made calibration platform that employs air inflatable bladder. The results showed that smaller variations in the interface pressures were found in pressure-cast socket, leading to a more consistently fitting socket. Pressure variations in PTB socket were steeper, resulting in increased shear stresses which are major causes of skin problems. Despite the different casting techniques, the interface pressures were higher in pressure-cast socket concept and not, as expected, in the PTB socket concept. Due to this discrepancy, the researchers could not confirm the uniform pressure distribution in the hydrostatic socket concept, highlighting that the interface pressure distribution exhibited a consistent pattern in the two socket types.

## 6. Effect of Liner Materials and Suspension Systems

**Figure 9:**
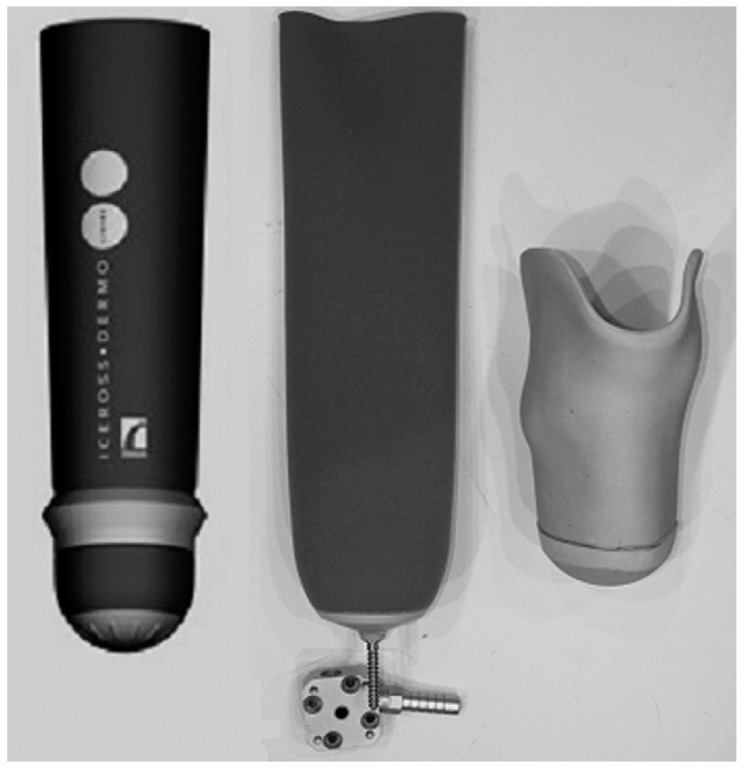
Three different liners, (**Left**) seal-in silicone liner; (**Middle**) pin/lock silicone liner; and (**Right**) Pelite liner [151].

Cushioning is crucial to protect the residual limb soft tissues as they are not accustomed to weight bearing [144,145]. Therefore, liners have been introduced to help in cushioning the transfer of loads and alleviating shock from the contact between the residual limb and prosthetic socket [30,146,147]. Until the early 1990s, transtibial amputees were equipped with PTB sockets with a soft polyethylene foam liner. Later in the mid-1990s, TSB sockets with silicone or elastomeric liners rolled onto the residual limb were proposed in an effort to offer better cushioning, suspension, and durability [147,148]. They also provide comfort and reduce shear forces [149]. However, sweating is the main problem encountering transtibial amputees using TSB sockets with silicone liners due to the reduced ventilation between the residual limb and liner. In addition, TSB socket users face difficulties in sitting due to the creases that appear at the popliteal fossa [150]. Therefore, amputees spending most of the day sitting prefer to use PTB sockets with the Pelite insert. Figure 9 shows various types of existing liners.

Silicone liners play an important role in securing the residual limb to the socket to provide good suspension. Pin and suction suspension systems are the most commonly used by amputees wearing gel liners within undersized TSB sockets, though the suspension methods are quite different [143]. The pin suspension employs a metal pin affixed at the distal end of the gel liner to keep it in place at the bottom of the socket. The suction suspension induces suction in the air space at the distal end of socket to prevent the liner from sliding proximally relative to the socket during the swing phase of gait cycle. Usually, a sleeve is used to seal the air space alongside a one-way valve at the distal socket.

To understand the effect of suspension systems on the comfort and satisfaction of amputees, Boonstra et al. conducted a study to compare the Fillauer silicone suction socket (3S) with shuttle locking mechanism to the supracondylar PTB socket with Pelite liner [152]. Of the eight subjects who participated in this study, two were not able to tolerate the 3S system and four preferred the use of Pelite system due to the ease of donning and doffing. Only the remaining two preferred the 3S system since it provides full contact of the residual limb. Another study by Coleman et al. assessed the overall ambulatory activity and subjects’ preference for amputees using an Alpha gel liner with distal pin/lock suspension and Pelite liners with neoprene suspension sleeves [17]. The questionnaire results showed that ten of the thirteen subjects participating in the study preferred to continue using Pelite liners. In addition, the subjects spent 82% more time wearing the Pelite and took 83% more steps per day. The aforementioned studies depended on subject’s preference and walking activity without recording the interface pressures within the subjects’ prosthetic sockets.

Until 2010, the literature reveals that despite the existence of many types of liners and suspension systems, there was little scientific evidence to inform the prescription practices of prosthetic liners. Instead, clinical prescription relied on the prosthetist experience, amputee feedback, and/or colleague recommendations [24,144]. Many researchers had previously been focusing on the mechanical properties of liner materials [153,154] and feedbacks from prosthesis wearers [17]. The mechanical properties have been summarized in review articles by Sanders et al. and Klute et al. [144,155]. Thereafter, Boutwell et al. have studied the effect of the gel liner thickness on the interface pressure between the residual limb and socket using the Pliance system [156]. They found that thicker liners significantly reduced the peak pressure at the fibula head in all subjects and can redistribute the loads more uniformly across the residual limbs. However, amputees with thicker liners experienced less stability during walking. While the material properties of liners had been well studied ex vivo, research discerning the significant differences in performance between different liners in situ to aid with clinical choices was lacking.

Recently, a series of research studies aiming at the assessment of different liners and suspension systems through the measurement of interface pressures between the residual limb and socket using Tekscan F-Socket transducers have been conducted at the University of Malaya CAB. Ali et al. have clinically investigated the interface pressure in TSB sockets with Dermo and Seal-In X5 liners during normal walking on level ground and their effect on patient satisfaction [136]. Nine unilateral amputees were involved in this study, each of whom was equipped with two sockets; one with Dermo liner with shuttle lock and another with Seal-In X5 liner with a valve. The Seal-In X5 liner has five seals at its distal part that conform to the shape of the internal socket wall and residual limb [157]. The F-Socket transducers were inserted between the residual limb and liner to measure pressures at the anterior, posterior, medial, and lateral aspects of the socket. The results of this investigation revealed that Dermo liner produces less interface peak pressures than those with Seal-In X5 liner. The mean peak pressures with Seal-In X5 liner were 34%, 24%, 7% higher at the anterior, posterior medial aspects of the socket (*p* = 0.008, *p* = 0.046, *p* = 0.025) than it was with Dermo liner. There was no significant difference between the liners at the lateral aspect of the socket. The amputees also felt more comfortable with the Dermo liner (*p* < 0.05). A qualitative study by the same research team compared the patients’ satisfaction and identified the perceived problems with the subjects’ prostheses while using three different suspension systems: Pelite, Dermo liner with shuttle lock, and Seal-In X5 liner [137]. Thirty patients with transtibial amputations agreed to participate in this study. The results of the survey confirmed the findings of the aforementioned clinical investigation of interface pressure using F-Socket transducers [136]. The patients demonstrated more satisfaction and fewer problems with Dermo liner compared to Pelite and Seal-In X5 liners. Therefore, it was concluded that the Dermo liner might be the best choice for transtibial amputees and these results might help the clinicians and prosthetists in selection criteria of prosthetic liners.

The research team expanded their investigations to compare the interface pressure between the two liners during more amputees’ daily activities such as stair ascent and decent [92] and ramp negotiation [138]. Ten amputees participated in this study and F-Socket mats were used to record the interface pressures. Again, the Dermo liner with shuttle lock showed lower interface pressures than Seal-In X5 liner at the anterior, posterior and medial aspects of the socket during stairs and ramp negotiation. In addition, their qualitative survey showed that the amputees wearing Seal-In X5 liner experienced less satisfaction and more encountered problems during stairs and ramp negotiation.

Eshraghi et al. patented a new magnetic-based coupling system capable of suspending the prosthesis with acoustic safety alarm system to ensure proper use with silicone liners [139]. They evaluated this system in-situ with regard to the pistoning during walking. The magnetic suspension system produced less pistoning compared to the pin/lock suspension system. The same researchers also used F-Socket transducers to experimentally investigate the interface pressures with the magnetic suspension system compared to the other two commonly used suspension systems: pin/lock and seal-in [32]. Twelve transtibial amputees volunteered to participate in this study and the resultant peak pressures with the three suspension systems were recorded during walking. There were statistically significant differences between the three systems. The magnetic system produced less peak pressures compared to the pin/lock system over the anterior and posterior aspects of the sockets. Furthermore, the highest interface pressures existed with the seal-in suspension system. Overall, the newly developed magnetic suspension system reduced the pressures within the socket, particularly during the swing phase of gait. It also reduced the pain and discomfort at distal end of the residual limb that is associated with the use of pin/lock suspension system. Eshraghi et al. also compared the effect of these three suspension systems on the interface pressures inside transtibial sockets during locomotion on stairs and ramps [140]. The greatest peak pressures were observed again with seal-in suspension system. It is obvious from the literature that magnetic system produced the lowest interface pressures inside the socket while the highest pressures exist with the seal-in system during walking, stair ascent and decent, and ramps. However, the pistoning is lessened with suction systems with Seal-in liner when compared to the other systems [150].

## 7. Conclusions

It is evident that interface normal and shear stresses in transtibial prosthetic sockets have been investigated using a variety of measurement systems developed during the past five decades. The use of such systems considerably helped researchers/prosthetists clearly understand the mechanical interactions between the residual limb and socket, thus contributed to the improvement of prosthetic socket designs. A few systems (i.e., Tekscan and Pliance) have been commercially available and could be efficacious in clinical settings, but they unfortunately are unable to measure shear stresses that are of great significance to researchers since they severely induce skin ulcers and irritations on the residual limb. On the other hand, SG-based transducers are capable of measuring both normal and shear stresses, but their bulkiness hampered their use in clinical settings. Unlike conventional transducers that are usually mounted on socket wall or inserted in the residual limb-socket interface, the emerging optical FBG and optoelectronic transducers could be embedded directly into the socket wall or silicone liner themselves, eliminating any measurement distortions that occur due to bulkiness and/or crease of conventional transducers. These superior advantages could make optical-based transducers become the alternative candidate to the aforementioned techniques. Yet, a collaborative work between engineers, scientists, and programming experts is highly recommended to bring these emerging optical-based techniques into maturity. As for the socket design concepts, the rapid prototyping manufacturing technique showed promising results such that they reduce the socket fabrication time from days to hours, affecting the cost and eliminating the tedious and laborious steps of traditional socket fabrication methods. Suspension systems that distally lock the silicone liner into socket (i.e., pin/lock or magnetic) developed the lowest interface pressures inside sockets and improved the overall satisfaction perceived by amputees.

## Figures and Tables

**Table 1:** Types of transducers used in transtibial prosthetic sockets and their relative merits and demerits.

Transducer Type	Ref.	Structure and Mounting Technique	Parameters to Measure	Merits	Demerits
Diaphragm SG (Kulite sensor)	[50,57]	(i) A circle-shaped sensing element with a diameter and thickness of 3.2 and 0.8 mm, respectively, and a four conductor ribbon cable of 0.5 mm thickness is attached to its bottom surface. (ii) It is a monolithic integrated circuit Wheatstone bridge formed directly on a silicone diaphragm. (iii) It could be inserted inside the socket. (iv) No longer used.	(i) Strains (ii) Forces (iii) Direct pressures	(i) Simplicity, (ii) High sensitivity, and (iii) Lightweight	(i) Its stiff backing mismatches with the residual limb tissues, causing stress concentrations at the sensor edges, (ii) Loads are measured at isolated sites, (iii) When put in an array of sensing elements. It would be subjected to crosstalk due to its rigidity and the cables restrict the subject movement which alters the amputee’s normal gait.
Piston-type SG	[42,45,58,59]	(i) Small patches of silicone or metal, (ii) An assembly of SG sensing elements and cylindrical piston are configured in a cylinder-like housing, and (iii) Mounted onto the socket wall in locations of significance through drilled holes	(i) Forces, and (ii) Normal & shear stresses	(i) High sensitivity and accuracy, (ii) No crosstalk and edge stress concentrations.	(i) Holes in the socket wall alter the pressure distribution, (ii) Bulky size, (iii) The data cables increased the prosthesis weight, distorting the stress measurement. (iv) Require a relatively more power to operate.
Single-point FSRs	[60,61]	(i) A sensitive element in form of an elastomer, conductive ink, conductive rubber, or carbon fiber that is sandwiched between two layers of flexible polyester films glued by an adhesive to form a piezoresistive pressure sensor. (ii) Positioned in-situ inside the prosthetic socket	(i) Forces, (ii) Direct contact pressures	(i) Thin construction, (ii) Small profile, (iii) Flexibility, (iv) Good sensitivity, (v) Relatively simple structure, and (vi) Ease of use. (vii) Available in various shapes and sizes	(i) Covers a very small sensing surface
Array of Piezoresistive	[60,62,63,64,65,66,67]	(i) Constructed of 96 individual sensing points (sensels) arranged in a matrix of 16 rows and 6 columns. (ii) Could be inside prosthetic sockets.	(i) Direct contact pressures	(i) Requires no modifications in sockets, making them superior over piston-type SGs (ii) Most commonly used piezoresistive sensing sheets for interface pressure measurement inside prosthetic sockets. (iii) Provides higher spatial resolution. (iv) Satisfactory reproducibility and sensitivity, (v) Flexibility, and (vi) Thin structure. (vii) Simple electronics	(i) Non-linearity (ii) Needs to be equilibrated and calibrated before each use, (iii) Drift, (iv) Hysteresis, (v) Temperature sensitivity. (vi) Their disability to measure shear stresses.
Capacitive (Single sensing element)	[68,69,70,71,72,73]	(i) A dielectric material sandwiched between two parallel conductive surfaces. (ii) Could be mounted inside and/or outside transtibial sockets.	(i) Forces (ii) Pressures (iii) Displacement	(i) Flexibility (ii) The operational accuracy was ±20%	(i) Their use in prosthetic sockets was limited due to their rigid substrates that do not comply with the residual limb geometry. (ii) Their sophisticated manufacturing techniques hindered low cost fabrication of multiple sensor arrays.
“Novel” Capacitive (Array)	[67,74]	(i) A matrix array of 16 sensing sites (4 × 4) mounted in silicone substrate (2.5 cm × 2.5 cm) with a thickness of 0.63 mm. (ii) Could be inserted between the skin and liner or between the liner and socket.	(i) Interface pressures	(i) Showed no noticeable sensor drift occurred between pre- and post-test calibration. (ii) Acceptable reliability and accuracy, and (iii) Superior to piezoresistive sensors	(i) Still unidirectional, measuring only direct pressures
3-D printed Capacitive	[1]	(i) A flexible frame (20 mm × 20 mm), with thickness of 4 mm. (ii) Could be inside prosthetic sockets	(i) Interface normal & shear stresses	(i) Low-cost and versatile solution with capability of adopting irregular shapes. (ii) Small in size (iii) Higher sensitivity and flexibility, lower temperature dependency, more robust structure, lower power consumption, better frequency response and a larger dynamic range than piezoresistive devices.	(i) Their susceptibility to crosstalk noise, (ii) Require more sophisticated electronics
Fibre-optics	[3,75]	(i) The optical fiber based sensors (FBG) has a longitudinal periodic variation of the refractive index n_eff_ written in the core of optical fiber for generating the required spatial pattern. When an optical fiber with an FBG is coupled to a light source and subjected to any external mechanical forces, the light passing through it will be back-reflected by the FBG itself at a Bragg wavelength, λ_B_, depending on the spacing between the periodic variations and the strain-optic effect. (ii) Could be inserted inside sockets, embedded in the socket wall, or embedded in the prosthetic silicone liners	(i) Strains, (ii) Forces, (iii) Normal & shear stresses, (iv) Vibration, (v) Temperature, etc.	(i) High sensitivity, (ii) durability, (iii) immunity to electromagnetic interference (EMI), (iv) mutiplexability, (v) and resistant to harsh environments	(i) Full operation might be hampered due to any damage to the optical fiber.
Optoelectronic	[76,77]	(i) Made of an external silicone bulk structure and a printed circuit board which accommodates an array of sensitive elements (LEDs & Photodiodes). (ii) Could be inserted inside sockets or embedded in the prosthetic silicone liners	(i) Normal and shear stresses, (ii) Displacement	(i) Accuracy (ii) Sensitivity	(i) Susceptible to EMIs (ii) Bulky

**Table 2:** Pressure transducers used within transtibial sockets based on different transduction methods.

Authors	Year	Objectives	Sensor Type	Mounting Method	Socket Type	Sites of Interest	No. of Subjects	Ref.
Rae and Cockrell	1971	To compare the differences in interface peak pressures in sockets with no liner, sponge liner, and silicone liner at that time.	Diaphragm SG (Kulite)	Inserted in socket	PTB	Condylar flairs (MTC, LTC), PT, distal anterior region (Kick-point, KP)	-	[57]
Pearson et al.	1973	To compare interface pressures during standing and walking.	Diaphragm SG (Kulite)	Inserted in socket	PTB	PT, KP, MTC, and LTC	10	[128]
Chino et al.	1975	To investigate the effect of various suspension systems on the suction pressure between the apex of the stump and the socket during the swing phase.	Diaphragm SG (Kulite)	Inserted in socket	PTB	KP	8	[23]
Sanders et al.	1990	To design the instrumentation capable of measuring normal and shear stresses simultaneously in prosthetic sockets	Piston-type SG	Mounted on socket wall	-	-	-	[129]
Sanders et al.	1993	To report the characteristics of interface stress wave-form shapes and their effects on stump tissue mechanics	Piston-type SG	Mounted on socket wall	PTB	At discrete points at all socket aspects	3	[130]
Sanders et al.	1997	To investigate the magnitudes of maximal stance phase pressure, maximal shear stress, shear angle and changes in pressures for each of the 13 sites in sockets of two amputees.	Piston-type SG	Mounted on socket wall	PTB	13 sites (anterior, lateral, and posterior)	2	[131]
Goh et al.	2003	To investigate pressure distribution in sockets fabricated using pressure casting (PCast) technique.	Piston-type SG	Mounted on socket wall	Hydrocast socket	16 Discrete points	5	[44]
Goh et al.	2004	To compare pressure profile of PCast and PTB sockets	Piston-type SG	Mounted on socket wall	PTB & Hydrocast	16 Discrete points	4	[132]
Abu Osman et al.	2010	To investigate the effect of varying the load (through the depth of indentation) on the patellar tendon bar on the pattern of pressure distribution at the stump–socket interface and if there is any correlation between varying the load on the patellar tendon and the pressure distribution at other sites in the socket	Piston-type SG	Mounted on socket wall	PTB	16 sites including those in high curvature regions	10	[79]
Meier et al.	1973	To investigate pressures on the residual limbs of 8 transtibial amputees.	Capacitive	Inserted in socket	PTB	5 sites	8	[73]
Dou et al.	2006	To measure pressures at five interesting sites of only one below-knee amputee socket during walking on stairs, flat, and non-flat roads	Capacitive	Inserted in socket		5 sites	1	[6]
Convery & Buis	1998, 1999	To compare the dynamic residual limb-socket interface pressure distributions in PTB and Hydrocast (TSB) sockets	Piezoresistive (F-Socket)	Attached to inner socket wall	PTB & Hydrocast	Overall impression of the interface	1	[133,134]
Dumbleton et al.	2009	To compare the dynamic interface pressure distribution and patient satisfaction between PTB sockets with Pelite liners and hydrostatic sockets with silicone liners	Piezoresistive (F-Socket)	Attached to inner socket wall	PTB & Hydrocast	Overall impression of the interface	48	[135]
Ali et al.	2012	To clinically investigate the interface pressure in TSB sockets with Dermo and Seal-In X5 liners during normal walking on level ground and their effect on patient satisfaction	Piezoresistive (F-Socket)	Attached in between the stump and liner	TSB	Overall impression of the interface	9	[136]
Ali et al.	2014	To compare the patients’ satisfaction and identify the perceived problems with the subjects’ prostheses while using three different suspension systems: Pelite, Dermo liner with shuttle lock, and Seal-In X5 liner	Piezoresistive (F-Socket)	Attached in between the stump and liner	TSB	Overall impression of the interface	30	[137]
Ali et al.	2013, 2015	To compare the interface pressure between the Dermo and Seal-In X5 liners during more amputees’ daily activities such as stair ascent and decent and ramp negotiation	Piezoresistive (F-Socket)	Attached in between the stump and liner	TSB	Overall impression of the interface	10	[92,138]
Eshraghi et al.	2013	To evaluate a patented magnetic-based suspension system in-situ with regard to the pistoning during walking	Piezoresistive (F-Socket)	Attached in between the stump and liner	TSB	Overall impression of the interface	-	[139]
Eshraghi et al.	2013	To experimentally investigate the interface pressures with the magnetic suspension system compared to the other two commonly used suspension systems: pin/lock and seal-in	Piezoresistive (F-Socket)	Attached in between the stump and liner	TSB	Overall impression of the interface	12	[32]
Eshraghi et al.	2015	To compare the effect of these three suspension systems on the interface pressures inside transtibial sockets during locomotion on stairs and ramps	Piezoresistive (F-Socket)	Attached in between the stump and liner	TSB	Overall impression of the interface	-	[140]

## Abbreviations

The following abbreviations are used in this manuscript:

PTB	Patellar Tendon Bearing
TSB	Total Tendon Bearing
PT	Patellar Tendon
SCSP	Supracondylar Suprapatellar
SC	Supracondylar
RP	Rapid Prototyping
CAD/CAM	Computer-Aided Design/Computer-Aided Manufacturing
2/3D	Two/Three Dimensional
SG	Strain Gauge
F-Socket	Force-Socket
FOS	Fiber Optical Sensor
FBG	Fiber Bragg Grating
PFBG	Polymer Fiber Bragg Grating
EMI	Electromagnetic Interference
GF	Gauge Factor
FSR	Force Sensing Resistor
PCB	Printed Circuit Board
LED	Light Emitting Diode
PCast	Pressure Casting
ANN	Artificial Neural Network
MDPI	Multidisciplinary Digital Publishing Institute
DOAJ	Directory of open access journals

## References

[1] Laszczak P., Jiang L., Bader D.L., Moser D., Zahedi S. (2015). Development and validation of a 3D-printed interfacial stress sensor for prosthetic applications. Med. Eng. Phys..

[2] Pirouzi G., Abu Osman N., Eshraghi A., Ali S., Gholizadeh H., Wan Abas W. (2014). Review of the socket design and interface pressure measurement for transtibial prosthesis. Sci. World J..

[3] Al-Fakih E., Osman A., Azuan N., Mahamd Adikan F., Eshraghi A., Jahanshahi P. (2016). Development and validation of fiber bragg grating sensing pad for interface pressure measurements within prosthetic sockets. IEEE Sens. J..

[4] Laing S., Lee P.V., Goh J.C. (2011). Engineering a trans-tibial prosthetic socket for the lower limb amputee. Ann. Acad. Med. Singap..

[5] Reiber G.E. (1994). Who is at risk of limb loss and what to do about it?. J. Rehabil. Res. Dev..

[6] Dou P., Jia X., Suo S., Wang R., Zhang M. (2006). Pressure distribution at the stump/socket interface in transtibial amputees during walking on stairs, slope and non-flat road. Clin. Biomech..

[7] Sanders J. (1995). Interface mechanics in external prosthetics: Review of interface stress measurement techniques. Med. Biol. Eng. Comput..

[8] Silver-Thorn M.B., Steege J.W., Childress D.S. (1996). A review of prosthetic interface stress investigations. J. Rehabil. Res. Dev..

[9] Polliack A., Sieh R., Craig D., Landsberger S., McNeil D., Ayyappa E. (2000). Scientific validation of two commercial pressure sensor systems for prosthetic socket fit. Prosthet. Orthot. Int..

[10] Zhang M., Turner-Smith A., Tanner A., Roberts V. (1998). Clinical investigation of the pressure and shear stress on the trans-tibial stump with a prosthesis. Med. Eng. Phys..

[11] Hafner B.J., Sanders J.E. (2014). Considerations for development of sensing and monitoring tools to facilitate treatment and care of persons with lower limb loss. J. Rehabil. Res. Dev..

[12] Radcliffe C.W., Foort J. (1961). The Patellar-Tendon-Bearing Below-Knee Prosthesis.

[13] Wu C.L., Chang C.H., Hsu A.T., Lin C.C., Chen S.I., Chang G.L. (2003). A proposal for the pre-evaluation protocol of below-knee socket design-integration pain tolerance with finite element analysis. J. Chin. Inst. Eng..

[14] Radcliffe C.W. (1962). The biomechanics of below-knee prostheses in normal, level, bipedal walking. Artif. Limbs.

[15] Ng P., Lee P., Goh J. (2002). Prosthetic sockets fabrication using rapid prototyping technology. Rapid Prototyp. J..

[16] Abu Osman N.A., Spence W.D., Solomonidis S.E., Paul J.P., Weir A.M. (2010). The patellar tendon bar! Is it a necessary feature?. Med. Eng. Phys..

[17] Coleman K.L., Boone D.A., Laing L.S., Mathews D.E., Smith D.G. (2004). Quantification of prosthetic outcomes: Elastomeric gel liner with locking pin suspension versus polyethylene foam liner with neoprene sleeve suspension. J. Rehabil. Res. Dev..

[18] Sewell P., Noroozi S., Vinney J., Andrews S. (2000). Developments in the trans-tibial prosthetic socket fitting process: A review of past and present research. Prosthet. Orthot. Int..

[19] Papaioannou G., Tsiokos D., Fiedler G., Mitrogiannis C., Avdeev I., Wood J., McKinney R. (2011). Dynamic radiography imaging as a tool in the design and validation of a novel intelligent amputee socket. Computational Vision and Medical Image Processing: Recent Trends.

[20] Breakey J.W. (1973). Criteria for use of supracondylar and supracondylar suprapatellar suspension for below-knee prostheses. Prosthet. Orthot. Int..

[21] Taft C. (1968). The patellar-tendon-supracondylar (pts) prosthesis: Report of a preliminary study. Inter-Clin. Inform. Bull..

[22] Girling J., Cummings G. (1972). Artificial-limb fabrication without the use of commercially available components. Prosthet. Orthot. Int..

[23] Chino N., Pearson J., Cockrell J., Mikishko H., Koepke G. (1975). Negative pressures during swing phase in below-knee prostheses with rubber sleeve suspension. Arch. Phys. Med. Rehabil..

[24] Baars E., Geertzen J. (2005). Literature review of the possible advantages of silicon liner socket use in trans-tibial prostheses. Prosthet. Orthot. Int..

[25] Safari M.R., Meier M.R. (2015). Systematic review of effects of current transtibial prosthetic socket designs—Part 1: Qualitative outcomes. J. Rehabil. Res. Dev..

[26] Galdik J. (1955). The below knee suction socket. Orthop. Prosthet. Appl. J..

[27] Friel K. (2005). Componentry for lower extremity prostheses. J. Am. Acad. Orthop. Surg..

[28] Foort J. (1965). The patellar-tendon-bearing prosthesis for below-knee amputees, a review of technique and criteria. Artif. Limbs.

[29] Yigiter K., Sener G., Bayar K. (2002). Comparison of the effects of patellar tendon bearing and total surface bearing sockets on prosthetic fitting and rehabilitation. Prosthet. Orthot. Int..

[30] Kristinsson Ö. (1993). The iceross concept: A discussion of a philosophy. Prosthet. Orthot. Int..

[31] Fergason J., Smith D.G. (1999). Socket considerations for the patient with a transtibial amputation. Clin. Orthop. Relat. Res..

[32] Eshraghi A., Abu Osman N.A., Gholizadeh H., Ali S., Sævarsson S.K., Abas W.A.B.W. (2013). An experimental study of the interface pressure profile during level walking of a new suspension system for lower limb amputees. Clin. Biomech..

[33] Edwards M.L. (2000). Below knee prosthetic socket designs and suspension systems. Phys. Med. Rehabil. Clin. N. Am..

[34] Eshraghi A., Abu Osman N.A., Gholizadeh H., Karimi M., Ali S. (2012). Pistoning assessment in lower limb prosthetic sockets. Prosthet. Orthot. Int..

[35] Pirouzi G., Abu Osman N.A., Oshkour A.A., Ali S., Gholizadeh H., Wan Abas W.A. (2014). Development of an air pneumatic suspension system for transtibial prostheses. Sensors.

[36] Eshraghi A., Abu Osman N.A., Karimi M.T., Gholizadeh H., Ali S., Abas W.A.B.W. (2012). Quantitative and qualitative comparison of a new prosthetic suspension system with two existing suspension systems for lower limb amputees. Am. J. Phys. Med. Rehabil..

[37] Tan K.C., Lee P.V.S., Tam K.F., Lye S.L. Automation of prosthetic socket design and fabrication using computer aided design/computer aided engineering and rapid prototyping techniques. Proceedings of the 1st National Symposium of Prosthetics and Orthotics.

[38] Comotti C., Regazzoni D., Rizzi C., Vitali A. Multi-material design and 3D printing method of lower limb prosthetic sockets. Proceedings of the 3rd 2015 Workshop on ICTs for improving Patients Rehabilitation Research Techniques.

[39] Faustini M.C., Neptune R.R., Crawford R.H., Rogers W.E., Bosker G. (2006). An experimental and theoretical framework for manufacturing prosthetic sockets for transtibial amputees. IEEE Trans. Neural Syst. Rehabil. Eng..

[40] Rogers B., Bosker G., Faustini M., Walden G., Neptune R.R., Crawford R. (2008). Case report: Variably compliant transtibial prosthetic socket fabricated using solid freeform fabrication. J. Prosthet. Orthot..

[41] Sengeh D.M., Herr H. (2013). A variable-impedance prosthetic socket for a transtibial amputee designed from magnetic resonance imaging data. J. Prosthet. Orthot..

[42] Appoldt F., Bennett L., Contini R. (1968). Stump-socket pressure in lower extremity prostheses. J. Biomech..

[43] Zachariah S., Sanders J. (2001). Standing interface stresses as a predictor of walking interface stresses in the trans-tibial prosthesis. Prosthet. Orthot. Int..

[44] Goh J., Lee P., Chong S. (2003). Stump/socket pressure profiles of the pressure cast prosthetic socket. Clin. Biomech..

[45] Sanders J.E., Daly C.H. (1993). Measurement of stresses in three orthogonal directions at the residual limb-prosthetic socket interface. IEEE Trans. Rehabil. Eng..

[46] Lee V., Solomonidis S., Spence W. (1997). Stump-socket interface pressure as an aid to socket design in prostheses for trans-femoral amputees—A preliminary study. Proc. Inst. Mech. Eng. H J. Eng. Med..

[47] Amali R., Noroozi S., Vinney J., Sewell P., Andrews S. (2006). Predicting interfacial loads between the prosthetic socket and the residual limb for below-knee amputees—A case study. Strain.

[48] Sahandi R., Sewell P., Noroozi S., Hewitt M. (2012). Remote monitoring of lower-limb prosthetic socket fit using wireless technologies. J. Med. Eng. Technol..

[49] Sewell P., Noroozi S., Vinney J., Amali R., Andrews S. (2012). Static and dynamic pressure prediction for prosthetic socket fitting assessment utilising an inverse problem approach. Artif. Intell. Med..

[50] Sonck W.A., Cockrell J.L., Koepke G.H. (1970). Effect of liner materials on interface pressures in below-knee prostheses. Arch. Phys. Med. Rehabil..

[51] Frieden J., Cugnoni J., Botsis J., Gmür T., Ćorić D. (2010). High-speed internal strain measurements in composite structures under dynamic load using embedded fbg sensors. Compos. Struct..

[52] Massaroni C., Saccomandi P., Schena E. (2015). Medical smart textiles based on fiber optic technology: An overview. J. Funct. Biomater..

[53] Zhang X., Yang L. (2014). A fiber bragg grating quasi-distributed sensing network with a wavelength-tunable chaotic fiber laser. Syst. Sci. Control Eng. Open Access J..

[54] Tiwana M.I., Redmond S.J., Lovell N.H. (2012). A review of tactile sensing technologies with applications in biomedical engineering. Sens. Actuators A Phys..

[55] Poeggel S., Tosi D., Duraibabu D., Leen G., McGrath D., Lewis E. (2015). Optical fibre pressure sensors in medical applications. Sensors.

[56] Ştefănescu D.M. (2011). Wheatstone bridge-the basic circuit for strain gauge force transducers. Handbook of Force Transducers.

[57] Rae J.W., Cockrell J.L. (1971). Interface pressure and stress distribution in prosthetic fitting. Bull. Prosthet. Res..

[58] Williams R., Porter D., Roberts V., Regan J. (1992). Triaxial force transducer for investigating stresses at the stump/socket interface. Med. Biol. Eng. Comput..

[59] Appoldt F.A., Bennett L., Contini R. (1970). Tangential pressure measurements in above-knee suction sockets. Bull. Prosthet. Res..

[60] Almassri A.M., Wan Hasan W., Ahmad S., Ishak A., Ghazali A., Talib D., Wada C. (2015). Pressure sensor: State of the art, design, and application for robotic hand. J. Sens..

[61] Schofield J.S., Evans K.R., Hebert J.S., Marasco P.D., Carey J.P. (2016). The effect of biomechanical variables on force sensitive resistor error: Implications for calibration and improved accuracy. J. Biomech..

[62] Luo Z.-P., Berglund L.J., An K.-N. (1998). Validation of f-scan pressure sensor system: A technical note. J. Rehabil. Res. Dev..

[63] Engsberg J., Springer J., Harder J. (1992). Quantifying interface pressures in below-knee-amputee sockets. Assoc. Child. Prosth. Orthot. Clin..

[64] Houston V., Mason C., LaBlanc K., Beatties A., Garbarini M., Lorenze E. Preliminary results with the DVA-tekscan BK prosthetic socket/residual limb stress measurement system. Proceedings of the 20th Annual Meeting American Academy of Orthotist and Prosthetist.

[65] Buis A., Convery P. (1997). Calibration problems encountered while monitoring stump/socket interface pressures with force sensing resistors: Techniques adopted to minimise inaccuracies. Prosthet. Orthot. Int..

[66] Hachisuka K., Takahashi M., Ogata H., Ohmine S., Shitama H., Shinkoda K. (1998). Properties of the flexible pressure sensor under laboratory conditions simulating the internal environment of the total surface bearing socket. Prosthet. Orthot. Int..

[67] Polliack A., Craig D., Sieh R., Landsberger S., McNeal D. (2002). Laboratory and clinical tests of a prototype pressure sensor for clinical assessment of prosthetic socket fit. Prosthet. Orthot. Int..

[68] Ruda E.M., Sanchez O.F.A., Mejia J.C.H., Gomez S.J., Flautero O.I.C. Design process of mechatronic device for measuring the stump stresses on a lower limb amputee. Proceedings of the 22nd Intl Congress of Mechanical Engineering (COBEM 2013).

[69] Lai C.H., Li-Tsang C.W. (2009). Validation of the pliance x system in measuring interface pressure generated by pressure garment. Burns.

[70] Wolf S.I., Alimusaj M., Fradet L., Siegel J., Braatz F. (2009). Pressure characteristics at the stump/socket interface in transtibial amputees using an adaptive prosthetic foot. Clin. Biomech..

[71] Safari M.R., Tafti N., Aminian G. (2015). Socket interface pressure and amputee reported outcomes for comfortable and uncomfortable conditions of patellar tendon bearing socket: A pilot study. Assist. Technol..

[72] Tiwana M.I., Shashank A., Redmond S.J., Lovell N.H. (2011). Characterization of a capacitive tactile shear sensor for application in robotic and upper limb prostheses. Sens. Actuators A Phys..

[73] Meier R., Meeks E., Herman R. (1973). Stump-socket fit of below-knee prostheses: Comparison of three methods of measurement. Arch. Phys. Med. Rehabil..

[74] Sundara-Rajan K., Bestick A., Rowe G., Klute G., Ledoux W., Wang H., Mamishev A. (2012). An interfacial stress sensor for biomechanical applications. Meas. Sci. Technol..

[75] Al-Fakih E., Abu Osman N.A., Mahamd Adikan F.R. (2012). The use of fiber bragg grating sensors in biomechanics and rehabilitation applications: The state-of-the-art and ongoing research topics. Sensors.

[76] De Rossi S., Lenzi T., Vitiello N., Donati M., Persichetti A., Giovacchini F., Vecchi F., Carrozza M.C. Development of an in-shoe pressure-sensitive device for gait analysis. Proceedings of the Annual International Conference of the IEEE Engineering in Medicine and Biology Society, EMBC.

[77] Lincoln L.S., Quigley M., Rohrer B., Salisbury C., Wheeler J. An optical 3d force sensor for biomedical devices. Proceedings of the 4th IEEE RAS & EMBS International Conference on Biomedical Robotics and Biomechatronics (BioRob).

[78] Bae J., An T., Kim Y., Ryu C. Analysis of digital load cell using 2.4 ghz band’s zig-bee. Proceedings of the 3rd IEEE Conference on Industrial Electronics and Applications.

[79] Abu Osman N.A., Spence W.D., Solomonidis S.E., Paul J.P., Weir A.M. (2010). Transducers for the determination of the pressure and shear stress distribution at the stump—Socket interface of trans-tibial amputees. Proc. Inst. Mech. Eng. Part B J. Eng. Manuf..

[80] Sanders J., Zachariah S., Jacobsen A., Fergason J. (2005). Changes in interface pressures and shear stresses over time on trans-tibial amputee subjects ambulating with prosthetic limbs: Comparison of diurnal and six-month differences. J. Biomech..

[81] Sanders J., Fergason J., Zachariah S., Jacobsen A. (2002). Case study: Interface pressure and shear stress changes with amputee weight loss: Case studies from two trans-tibial amputee subjects. Prosthet. Orthot. Int..

[82] Mak A.F., Zhang M., Boone D.A. (2001). State-of-the-art research in lower-limb prosthetic biomechanics-socket interface. J. Rehabil. Res. Dev..

[83] Goh J., Lee P., Chong S. (2003). Static and dynamic pressure profiles of a patellar-tendon-bearing (PTB) socket. Proc. Inst. Mech. Eng. H J. Eng. Med..

[84] Frossard L., Beck J., Dillon M., Evans J. (2003). Development and preliminary testing of a device for the direct measurement of forces and moments in the prosthetic limb of transfemoral amputees during activities of daily living. J. Prosthet. Orthot..

[85] Hollinger A., Wanderley M.M. Evaluation of commercial force-sensing resistors. Proceedings of the International Conference on New Interfaces for Musical Expression.

[86] Kane B.J., Cutkosky M.R., Kovacs G.T. (2000). A traction stress sensor array for use in high-resolution robotic tactile imaging. J. Microelectromech. Syst..

[87] Park W.-T., Mallon J.R., Rastegar A.J., Pruitt B.L. (2009). Review: Semiconductor piezoresistance for microsystems. IEEE Proc..

[88] Saccomandi P., Schena E., Oddo C.M., Zollo L., Silvestri S., Guglielmelli E. (2014). Microfabricated tactile sensors for biomedical applications: A review. Biosensors.

[89] Vecchi F., Freschi C., Micera S., Sabatini A.M., Dario P., Sacchetti R. Experimental evaluation of two commercial force sensors for applications in biomechanics and motor control. Proceedings of the 5th Annual Conference of the International Functional Electrical Stimulation Society.

[90] Zhang H., So E. (2002). Hybrid resistive tactile sensing. IEEE Trans. Syst. Man Cybern. B Cybern..

[91] Shem K.L., Breakey J.W., Werner P.C. (1998). Pressures at the residual limb-socket interface in transtibial amputees with thigh lacer-side joints. J. Prosthet. Orthot..

[92] Ali S., Abu Osman N.A., Eshraghi A., Gholizadeh H., Abas W.A.B.B.W. (2013). Interface pressure in transtibial socket during ascent and descent on stairs and its effect on patient satisfaction. Clin. Biomech..

[93] Wolsley C., Hill P. (2000). Review of interface pressure measurement to establish a protocol for their use in the assessment of patient support surfaces. J. Tissue Viability.

[94] Zhou M.-X., Huang Q.-A., Qin M., Zhou W. (2005). A novel capacitive pressure sensor based on sandwich structures. J. Microelectromech. Syst..

[95] Razian M., Pepper M.G. (2003). Design, development, and characteristics of an in-shoe triaxial pressure measurement transducer utilizing a single element of piezoelectric copolymer film. IEEE Trans. Neural Syst. Rehabil. Eng..

[96] Hugenholtz P.G., Gamble W.J., Monroe G.R., Polanyi M. (1965). The use of fiberoptics in clinical cardiac catheterization ii. In vivo dye-dilution curves. Circulation.

[97] Roriz P., Frazão O., Lobo-Ribeiro A.B., Santos J.L., Simões J.A. (2013). Review of fiber-optic pressure sensors for biomedical and biomechanical applications. J. Biomed. Opt..

[98] Poeggel S., Duraibabu D., Kalli K., Leen G., Dooly G., Lewis E., Kelly J., Munroe M. (2015). Recent improvement of medical optical fibre pressure and temperature sensors. Biosensors.

[99] Rao Y.-J., Webb D.J., Jackson D.A., Zhang L., Bennion I. (1998). Optical in-fiber bragg grating sensor systems for medical applications. J. Biomed. Opt..

[100] Vo-Dinh T. (2014). Biomedical Photonics Handbook: Biomedical Diagnostics.

[101] Keiser G., Xiong F., Cui Y., Shum P.P. (2014). Review of diverse optical fibers used in biomedical research and clinical practice. J. Biomed. Opt..

[102] Mishra V., Singh N., Tiwari U., Kapur P. (2011). Fiber grating sensors in medicine: Current and emerging applications. Sens. Actuators A Phys..

[103] Rocha R., Gomes J., Carmo J., Silva A., Correia J. (2014). Low-cost/high-reproducibility flexible sensor based on photonics for strain measuring. Opt. Laser Technol..

[104] Fresvig T., Ludvigsen P., Steen H., Reikerås O. (2008). Fibre optic bragg grating sensors: An alternative method to strain gauges for measuring deformation in bone. Med. Eng. Phys..

[105] Yu Q., Zhou X. (2011). Pressure sensor based on the fiber-optic extrinsic fabry-perot interferometer. Photon. Sens..

[106] Liu X., Iordachita I.I., He X., Taylor R.H., Kang J.U. (2012). Miniature fiber-optic force sensor based on low-coherence fabry-pérot interferometry for vitreoretinal microsurgery. Biomed. Opt. Express.

[107] Bartelt H., Elsmann T., Habisreuther T., Schuster K., Rothhardt M. Optical bragg grating sensor fibers for ultra-high temperature applications. Proceedings of the 5th Asia Pacific Optical Sensors Conference.

[108] Gao R., Jiang Y., Ding W., Wang Z., Liu D. (2013). Filmed extrinsic fabry–perot interferometric sensors for the measurement of arbitrary refractive index of liquid. Sens. Actuators B Chem..

[109] Anwar Zawawi M., O’Keffe S., Lewis E. (2013). Intensity-modulated fiber optic sensor for health monitoring applications: A comparative review. Sens. Rev..

[110] Polygerinos P., Zbyszewski D., Schaeffter T., Razavi R., Seneviratne L.D., Althoefer K. (2010). Mri-compatible fiber-optic force sensors for catheterization procedures. IEEE Sens. J..

[111] Roriz P., Carvalho L., Frazão O., Santos J.L., Simões J.A. (2014). From conventional sensors to fibre optic sensors for strain and force measurements in biomechanics applications: A review. J. Biomech..

[112] Di Sante R. (2015). Fibre optic sensors for structural health monitoring of aircraft composite structures: Recent advances and applications. Sensors.

[113] Mihailov S.J. (2012). Fiber bragg grating sensors for harsh environments. Sensors.

[114] Udd E., Spillman W.B. (2011). Fiber Optic Sensors: An Introduction for Engineers and Scientists.

[115] Abushagur A.A., Arsad N., Reaz M.I., Bakar A. (2014). Advances in bio-tactile sensors for minimally invasive surgery using the fibre bragg grating force sensor technique: A survey. Sensors.

[116] Quandt B.M., Scherer L.J., Boesel L.F., Wolf M., Bona G.L., Rossi R.M. (2015). Body-monitoring and health supervision by means of optical fiber-based sensing systems in medical textiles. Adv. Healthc. Mater..

[117] Murukeshan V., Chan P., Ong L., Seah L. (2000). Cure monitoring of smart composites using fiber bragg grating based embedded sensors. Sens. Actuators A Phys..

[118] Kanellos G.T., Papaioannou G., Tsiokos D., Mitrogiannis C., Nianios G., Pleros N. (2010). Two dimensional polymer-embedded quasi-distributed fbg pressure sensor for biomedical applications. Opt. Express.

[119] Kanellos G.T., Tsiokos D., Pleros N., Papaioannou G., Childs P., Pissadakis S. Enhanced durability fbg-based sensor pads for biomedical applications as human-machine interface surfaces. Proceedings of the 2011 International Workshop on Biophotonics.

[120] Tsiokos D., Papaioannou G., Kanellos G.T., Pissadakis S. (2012). Fiber Optic-Based Pressure Sensing Surface for Skin Health Management in Prosthetic and Rehabilitation Interventions.

[121] Al-Fakih E.A., Osman N.A.A., Eshraghi A., Adikan F.R.M. (2013). The capability of fiber bragg grating sensors to measure amputees’ trans-tibial stump/socket interface pressures. Sensors.

[122] Zhang Z.F., Tao X.M., Zhang H.P., Zhu B. (2013). Soft fiber optic sensors for precision measurement of shear stress and pressure. IEEE Sens. J..

[123] De Rossi S.M.M., Vitiello N., Lenzi T., Ronsse R., Koopman B., Persichetti A., Vecchi F., Ijspeert A.J., Van der Kooij H., Carrozza M.C. (2011). Sensing pressure distribution on a lower-limb exoskeleton physical human-machine interface. Sensors.

[124] Donati M., Vitiello N., De Rossi S.M.M., Lenzi T., Crea S., Persichetti A., Giovacchini F., Koopman B., Podobnik J., Munih M. (2013). A flexible sensor technology for the distributed measurement of interaction pressure. Sensors.

[125] Missinne J., Bosnian E., Van Hoe B., Van Steenberge G., Van Daele P., Vanfleteren J. Embedded flexible optical shear sensor. Proceedings of the 2010 IEEE Sensors.

[126] Wheeler J. A Better Prosthesis: Sandia Invents Sensor to Learn About Fit; System to Make Fit Better. https://share.sandia.gov/news/resources/news_releases/prosthesic_sensor.

[127] Yousef H., Boukallel M., Althoefer K. (2011). Tactile sensing for dexterous in-hand manipulation in robotics—A review. Sens. Actuators A Phys..

[128] Pearson J.R., Holmgren G., March L., Oberg K. (1973). Pressures in critical regions of the below-knee patellar-tendon-bearing prosthesis. Bull. Prosthet. Res..

[129] Sanders J., Boone D., Daly C. The residual limb/prosthetic socket interface normal stress and shear stress. Proceedings of the 13th Annual RESNA Conference.

[130] Sanders J., Daly C., Burgess E. (1993). Clinical measurement of normal and shear stresses on a trans-tibial stump: Characteristics of wave-form shapes during walking. Prosthet. Orthot. Int..

[131] Sanders J.E., Lain D., Dralle A.J., Okumura R. (1997). Interface pressures and shear stresses at thirteen socket sites on two persons with transtibial amputation. J. Rehabil. Res. Dev..

[132] Goh J.C.H., Lee P.V.S., Chong S.Y. (2004). Comparative study between patellar-tendon-bearing and pressure cast prosthetic sockets. J. Rehabil. Res. Dev..

[133] Convery P., Buis A. (1998). Conventional patellar-tendon-bearing (PTB) socket/stump interface dynamic pressure distributions recorded during the prosthetic stance phase of gait of a transtibial amputee. Prosthet. Orthot. Int..

[134] Convery P., Buis A. (1999). Socket/stump interface dynamic pressure distributions recorded during the prosthetic stance phase of gait of a trans-tibial amputee wearing a hydrocast socket. Prosthet. Orthot. Int..

[135] Dumbleton T., Buis A.W., McFadyen A., McHugh B.F., McKay G., Murray K.D., Sexton S. (2009). Dynamic interface pressure distributions of two transtibial prosthetic socket concepts. J. Rehabil. Res. Dev..

[136] Ali S., Osman N.A.A., Mortaza N., Eshraghi A., Gholizadeh H., Abas W.A.B.B.W. (2012). Clinical investigation of the interface pressure in the trans-tibial socket with dermo and seal-in x5 liner during walking and their effect on patient satisfaction. Clin. Biomech..

[137] Ali S., Abu Osman N.A., Arifin N., Gholizadeh H., Abd Razak N.A., Wan Abas W.A.B. (2014). Comparative study between dermo, pelite, and seal-in x5 liners: Effect on patient’s satisfaction and perceived problems. Sci. World J..

[138] Ali S., Abu Osman N.A., Razak A., Hussain S., Abas W. (2015). The effect of dermo and seal-in x5 prosthetic liners on pressure distributions and reported satisfaction during ramp ambulation in persons with transtibial limb loss. Eur. J. Phys. Rehabil. Med..

[139] Eshraghi A., Abu Osman N.A., Gholizadeh H., Ahmadian J., Rahmati B., Abas W.A.B.W. (2013). Development and evaluation of new coupling system for lower limb prostheses with acoustic alarm system. Sci. Rep..

[140] Eshraghi A., Abu Osman N.A., Gholizadeh H., Ali S., Abas W.A.B.W. (2015). Interface stress in socket/residual limb with transtibial prosthetic suspension systems during locomotion on slopes and stairs. Am. J. Phys. Med. Rehabil..

[141] Jia X., Suo S., Meng F., Wang R. (2008). Effects of alignment on interface pressure for transtibial amputee during walking. Disabil. Rehabil. Assist. Technol..

[142] Sanders J.E., Daly C.H. Normal and shear interface stresses in lower-limb prosthetics. Proceedings of the Annual International Conference of the IEEE Engineering in Engineering in Medicine and Biology Society.

[143] Beil T.L., Street G.M. (2004). Comparison of interface pressures with pin and suction suspension systems. J. Rehabil. Res. Dev..

[144] Klute G.K., Glaister B.C., Berge J.S. (2010). Prosthetic liners for lower limb amputees: A review of the literature. Prosthet. Orthot. Int..

[145] Dudek N.L., Marks M.B., Marshall S.C., Chardon J.P. (2005). Dermatologic conditions associated with use of a lower-extremity prosthesis. Arch. Phys. Med. Rehabil..

[146] Emrich R., Slater K. (1998). Comparative analysis of below-knee prosthetic socket liner materials. J. Med. Eng. Technol..

[147] Hatfield A., Morrison J. (2001). Polyurethane gel liner usage in the oxford prosthetic service. Prosthet. Orthot. Int..

[148] Van de Weg F., Van der Windt D. (2005). A questionnaire survey of the effect of different interface types on patient satisfaction and perceived problems among trans-tibial amputees. Prosthet. Orthot. Int..

[149] Cochrane H., Orsi K., Reilly P. (2001). Lower limb amputation part 3: Prosthetics-a 10 year literature review. Prosthet. Orthot. Int..

[150] Gholizadeh H., Abu Osman N.A., Eshraghi A., Ali S., Razak N.A. (2014). Transtibial prosthesis suspension systems: Systematic review of literature. Clin. Biomech..

[151] Ali S., Abu Osman N.A., Naqshbandi M.M., Eshraghi A., Kamyab M., Gholizadeh H. (2012). Qualitative study of prosthetic suspension systems on transtibial amputees’ satisfaction and perceived problems with their prosthetic devices. Arch. Phys. Med. Rehabil..

[152] Boonstra A., Van Duin W., Eisma W. (1996). Silicone suction socket (3S) versus supracondylar PTB prosthesis with pelite liner: Transtibial amputees’ preferences. J. Prosthet. Orthot..

[153] Sanders J.E., Daly C.H., Cummings W.R., Reed R.D., Robert J MARKS I. (1994). A measurement device to assist amputee prothetic fitting. J. Clin. Eng..

[154] Sanders J.E., Greve J.M., Mitchell S.B., Zachariah S.G. (1998). Material properties of commonly-used interface materials and their static coefficients of friction with skin and socks. J. Rehabil. Res. Dev..

[155] Sanders J.E., Nicholson B.S., Zachariah S.G., Cassisi D.V. (2004). Testing of elastomeric liners used in limb prosthetics: Classification of 15 products by mechanical performance. J. Rehabil. Res. Dev..

[156] Boutwell E., Stine R., Tucker K. (2012). Effect of prosthetic gel liner thickness on gait biomechanics and pressure distribution within the transtibial socket. J. Rehabil. Res. Dev..

[157] Gholizadeh H., Osman N.A.A., Kamyab M., Eshraghi A., Lúvíksdóttir Á.G., Abas W.A.B.W. (2012). Clinical evaluation of two prosthetic suspension systems in a bilateral transtibial amputee. Am. J. Phys. Med. Rehabil..

